# FABP4 in Paneth cells regulates antimicrobial protein expression to reprogram gut microbiota

**DOI:** 10.1080/19490976.2022.2139978

**Published:** 2022-10-31

**Authors:** Xiaomin Su, Mengli Jin, Chen Xu, Yunhuan Gao, Yazheng Yang, Houbao Qi, Qianjing Zhang, Xiaorong Yang, Wang Ya, Yuan Zhang, Rongcun Yang

**Affiliations:** a Department of Immunology, Nankai University School of Medicine; Nankai University, Tianjin, China; b Translational Medicine Institute, Affiliated Tianjin Union Medical Center of Nankai University, Tianjin, China; c State Key Laboratory of Medicinal Chemical Biology, Nankai University, Tianjin, China; d Department of Colorectal Surgery, Tianjin Union Medical Center, Tianjin, China

**Keywords:** FABP4, defensing, Paneth cells, PPARγ

## Abstract

Antimicrobial proteins possess a broad spectrum of bactericidal activity and play an important role in shaping the composition of gut microbiota, which is related to multiple diseases such as metabolic syndrome. However, it is incompletely known for the regulation of defensin expression in the gut Paneth cells. Here, we found that FABP4 in the Paneth cells of gut epithelial cells and organoids can downregulate the expression of defensins. FABP4^fl/fl^pvillin^CreT^ mice were highly resistance to *Salmonella* Typhimurium (*S*.T) infection and had increased bactericidal ability to pathogens. The FABP4-mediated downregulation of defensins is through degrading PPARγ after K48 ubiquitination. We also demonstrate that high-fat diet (HFD)-mediated downregulation of defensins is through inducing a robust FABP4 in Paneth cells. *Firmicutes*/*Bacteroidetes* (F/B) ratio in FABP4^fl/fl^pvillin^CreT^ mice is lower than control mice, which is opposite to that in mice fed HFD, indicating that FABP4 in the Paneth cells could reprogram gut microbiota. Interestingly, FABP4-mediated downregulation of defensins in Paneth cells not only happens in mice but also in human. A better understanding of the regulation of defensins, especially HFD-mediated downregulation of defensin in Paneth cells will provide insights into factor(s) underlying modern diseases.

**Abbreviations**: FABP4: Fatty acid binding protein 4; S. T: Salmonella Typhimurium; HFD: High-fat diet; Defa: α-defensin; 930 HD5: Human α-defensin 5; HD6: Human α-defensin 6; F/B: Firmicutes/Bacteroidetes; SFB: Segmental filamentous bacteria; AMPs: Antimicrobial peptides; PPARγ: Peroxisome proliferator-activated receptor γ; P-PPAR: Phosphorylated PPAR; Dhx15: DEAD-box helicase 15; 935 EGF: Epidermal growth factor; ENR: Noggin and R-spondin 1; CFU: Colony forming unit; Lyz1: Lysozyme 1; Saa1: Serum amyoid A 1; Pla2g2a: Phospholipase A2, group IIA; MMP-7: Matrix metalloproteinase; AU-PAGE: Acid-urea polyacrylamide gel electrophoresis; PA: Palmitic 940 acid; GPR40: G-protein-coupled receptor; GF: Germ-free; EGF: Epidermal growth factor; LP: Lamina propria; KO: Knock out; WT: Wild-type.

## Introduction

Intestinal epithelial cells mainly include enterocytes, goblet cells, Paneth cells, stem cells, and enteroendocrine cells. Paneth cells, which are located at the base of the crypts of Lieberkühn in the intestine, play a critical role in host homeostasis. ^1^ They can produce large quantities of defensins. ^2^ The defensins, including HD5 and HD6 in humans and cryptdins in mice are expressed exclusively in the Paneth cells of the epithelial cells in small intestines and colons. ^3^ They possess a broad spectrum of antimicrobial activity to Gram positive and negative bacteria, fungi, viruses, and unicellular parasites. ^4^ The defensins not only disorganize bacterial cell membranes ^5^ and create trapping nanonets around bacteria ^6^ but also inactivate bacterial toxins and viral proteins. Host defensins, secreted by colonic epithelial cells also are critical components of an innate immune response in the colon against enteropathogenic bacteria ^7^ such as human α-defensin 6. ^6^ The defensins are physiologically involved in shaping the composition of gut microbiome ^8^ and play a critical role in maintaining homeostasis of gut microbiota. ^7,9,10^ For example, reduced α-defensins in the enteric epithelium can result in segmental filamentous bacteria (SFB) dysbiosis. ^11^ Decreased expression of a group of defensins causes increased *Clostridium cluster* XIVa in colonic microbiota, which is capable of inducing T_reg_ cells. ^12^ Severe gut pathologies are also associated with disrupted antimicrobial production in Paneth cells, as observed in chronic inflammatory and infectious diseases. ^13–15^

The expression, secretion and activities of epithelial host antimicrobial peptides (AMPs) are tightly controlled by multiple positive and negative regulatory mechanisms. ^16^ AMPs in Paneth cells include α/β-defensins, lysozyme, Reg3s and lectins, ^17^ whose induction may be controlled by the TLR/Wnt pathways. ^18^ The peroxisome proliferator-activated receptor (PPAR)γ, a nuclear receptor is also involved in mucosal defense regulation. Colonic mucosa of *PPARγ* mutant animals shows defective killing of several major components of the intestinal microbiota, including *Candida albicans, Bacteroides fragilis, Enterococcus faecalis*, and *Escherichia coli*. ^19,20^ Defensin expression in Paneth cells can also be modulated by dietary factors, including fibers, lipids, polyphenols, and vitamins. ^21^ A western-style die (WSD), characterized by its low dietary fiber but high-fat and high carbohydrate content, markedly changes microbiota composition in humans and mice and leads to a downregulation of defensins. ^22–25^ *Su et al*. demonstrated that high-fat diet reduced the expression of defensins ^26^ and led to a downregulation of PPARγ. ^27^ These mice had an altered colonic microbiota composition that could cause increased penetrability and a reduced growth rate of the inner mucus layer. ^22^ However, the mechanism(s) for high-fat diets (HFD)-mediated downregulation of defensins remains undefined.

Fatty acid binding protein 4 (FABP4) is a member of the FABP family of intracellular lipid chaperones. ^28^ It is most abundantly expressed in adipocytes but also detected in macrophages and a subset of endothelial cells such as gut epithelial crypt cells. ^29–32^ FABP4 exhibits a range of functions in these cell types, including regulation of glucose, lipid metabolism, and inflammation, cell survival and cell proliferation. ^33,34^ Circulating FABP4 levels are also associated with several aspects of metabolic syndrome and cardiovascular disease. ^35^ FABP4 is a critical mediator of metabolism and inflammatory processes, both locally and systemically, and therefore is potential therapeutic target for immunometabolic diseases. ^36^ However, the exact function(s) of FABP4 in gut epithelial crypt cells such as Paneth cells ^30^ is unclear. Since Paneth cells can highly produce antimicrobials to control gut microbial communities, ^37^ it is interest to know whether FABP4 in gut Paneth cells could regulate the antimicrobial peptides. Here, we find that FABP4, which can be induced by HFD, downregulates the expression of defensins through degrading PPARγ in Paneth cells.

## Results

### FABP4 in Paneth cells does not affect development of gut epithelial cells

**Figure 1.**
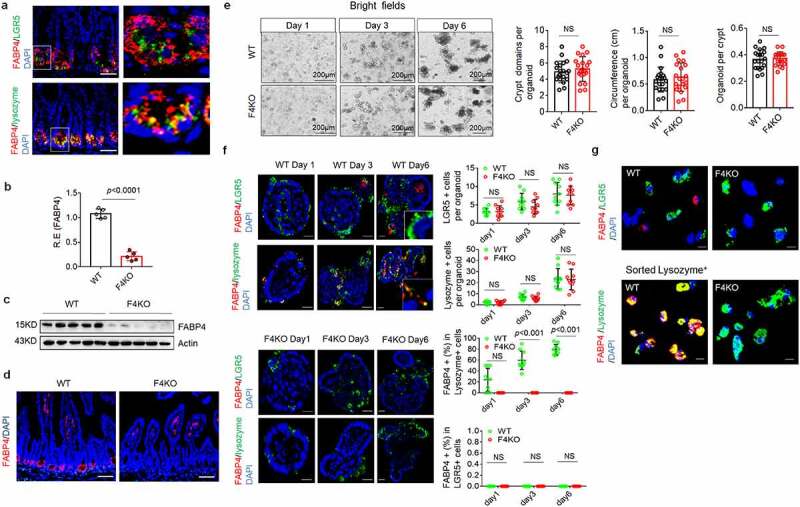
**FABP4 in Paneth cells does not affect development of gut epithelial cells**. (a) Immunostaining of FABP4 and LGR5 or lysozyme in the ileum tissues of mice. DAPI, nuclei staining. Scale bar = 40 μm. (b) QRT-PCR of FABP4 in the ileum tissues from FABP4^fl/fl^pvillin^CreT^ (F4KO) and FABP4^fl/fl^ (WT) mice (n = 5). R.E, relative expression. (c) Immunoblotting of FABP4 in the ileum tissues from FABP4^fl/fl^pvillin^CreT^ (F4KO) and FABP4^fl/fl^ (WT) mice (n = 5). (d) Immunostaining of FABP4 in the ileum from FABP4^fl/fl^pvillin^CreT^ (F4KO) and FABP4^fl/fl^ (WT) mice. DAPI, nuclei staining. Scale bar = 40 μm. (e) *In vitro* cultured gut organoids of FABP4^fl/fl^pvillin^CreT^ (F4KO) and FABP4^fl/fl^ (WT) mice on day 1, day 3 and day 6. (f) Immunostaining of FABP4 and LGR5 or lysozyme in the gut organoids of FABP4^fl/fl^pvillin^CreT^ (F4KO) and FABP4^fl/fl^ (WT) mice on day 1, day 3 or day 6. (g) Immunostaining of FABP4 and LGR5 or lysozyme in the isolated cells from the gut organoids of FABP4^fl/fl^pvillin^CreT^ (F4KO) and FABP4^fl/fl^ (WT) mice. *In vitro* cultured organoids were dissociated with TrypLE Express enzyme and DNase I into single cells at 37°C for 5 min. Sorting was done after staining using anti-lysozyme-FITC antibody. Scale bar = 20 μm. Student’s *t*-test, mean ± SD. NS, no significance.

FABP4 in gut epithelial crypt cells has been reported by us ^30^ and others, ^31,32^ and further confirmed in gut epithelial crypt cells (Figure 1a). To investigate the function(s) of FABP4 in gut epithelial crypt cells, we generated gut FABP4 conditional knockout mice (FABP4^fl/fl^pvillin^CreT^ mice) and control FABP4^fl/fl^ mice (Figure 1b-d). Previous reports indicated that interaction between Paneth cells and gut stem cells could affect development of gut epithelial cells. ^38^ However, there did not show difference in gut epithelial tissues between FABP4^fl/fl^pvillin^CreT^ mice and FABP4^fl/fl^ mice (Figure S1), implying that FABP4 does not affect development of gut epithelial cells. To further determine the effects of FABP4 on gut epithelial cells, we cultured *in vitro* gut organoids of FABP4^fl/fl^ mice and FABP4^fl/fl^pvillin^CreT^ mice. As previously reported, ^39,40^ small-intestinal crypts isolated from mice were embedded in Matrigel, and then cultured in the medium with epidermal growth factor (EGF), Noggin and R-spondin 1 (ENR). These gut organoids did not show difference between FABP4^fl/fl^ and FABP4^fl/fl^pvillin^CreT^ mice after *in vitro* culture for 6 days (Figure 1e, f). The gut cells in ENR could grow into organoids with intestinal epithelial cell types such as Paneth cells (lysozyme^+^) and LGR5^+^ stem cells (Figure 1f). Similar to previous reports, ^39,40^ these organoids behaved in a stereotypical manner. Their upper openings were sealed. The lumens were filled with apoptotic cells (Figure 1e). Total cell number, LGR5^+^stem cells and lysozyme^+^ Paneth cells did not show significantly difference between FABP4^fl/fl^ and FAPB4^fl/fl^pvillin^creT^ mice after *in vitro* culture for 6 days (Figure 1f), further suggesting that differentiation of gut cells does not be affected by FABP4. No FABP4^+^ cells were found in the gut organoids of FABP4^fl/fl^pvillin^CreT^ mice (Figure1f). *In vitro* cultured small intestinal organoids of FABP4^fl/fl^ mice showed that FABP4 was only expressed in lysozyme^+^ Paneth cells (Figure 1f, g). Thus, FABP4 in Paneth cells does not affect development and differentiation of gut epithelial cells.

### FABP4^fl/fl^pvillin^CreT^ mice have markedly increased bactericidal ability to pathogens

**Figure 2.**
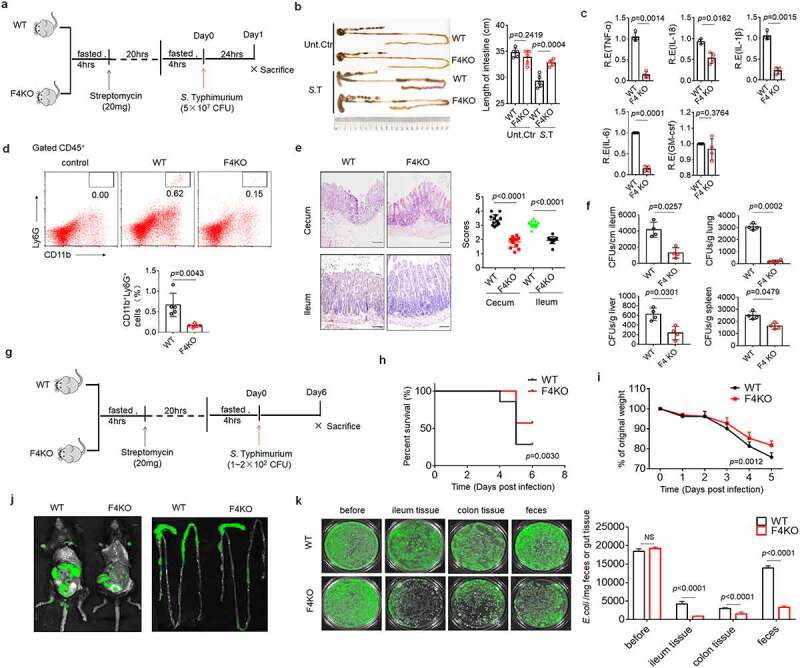
**FABP4^fl/fl^pvillin^CreT^ mice have markedly resistance against pathogen infection**. (a) Schematic of the experiment for the acute infection of *S*.T. (b) Length of the intestines and colons of FABP4^fl/fl^pvillin^CreT^ (F4KO) and FABP4^fl/fl^ (WT) mice with or without acute infection of *S*.T. (c) QRT-PCR of TNFα, IL-18, IL-6, IL-1β and GM-CSF in the ileum of FABP4^fl/fl^pvillin^CreT^ (F4KO) and FABP4^fl/fl^ (WT) mice with or without acute infection of *S*.T. R.E, relative expression. (d) Flow cytometry of CD11b^+^Ly6G^+^ cells in the ileum of FABP4^fl/fl^pvillin^CreT^ (F4KO) and FABP4^fl/fl^ (WT) mice with or without acute infection of *S*.T. (e) H/E staining of the ileum and cecum of FABP4^fl/fl^pvillin^CreT^ (F4KO) and FABP4^fl/fl^ (WT) mice with or without acute infection of *S*.T. (f) CFUs of *S*.T in the ileum, lung, spleen and liver of FABP4^fl/fl^pvillin^CreT^ (F4KO) and FABP4^fl/fl^ (WT) mice with or without acute infection of *S*.T. Equal weight tissues were homogenized in equal of amount of PBS. Homogenates were serially diluted and plated on Salmonella chromogenic agar to quantify CFUs of *S*.T. (g) Schematic of the experiment for the chronic infection of *S*.T. (h) Survival rate of FABP4^fl/fl^pvillin^CreT^ (F4KO) and FABP4^fl/fl^ (WT) mice with chronic infection of *S*.T (n = 12). (i) Body weights of FABP4^fl/fl^pvillin^CreT^ (F4KO) and FABP4^fl/fl^ (WT) mice with chronic infection of *S*.T (n = 12). (j) Fluorescence intensity of GFP-labelled *E.coli* in FABP4^fl/fl^pvillin^CreT^ (F4KO) and FABP4^fl/fl^ (WT) mice after infusing GFP-labelled *E. coli*. (k) CFUs of GFP-labelled *E. coli* in the ileum, colon and feces of FABP4^fl/fl^pvillin^CreT^ (F4KO) and FABP4^fl/fl^ (WT) mice after infection for 3 days. Tissues or contents were weighted, and then serially diluted and plated on LB agar for *E.coli* (n = 3). Student’s *t*-test in b, c, d, e, f and k; Wilcoxon’s test in H; Analysis of variance test in i. *P < 0.05, **P < 0.01, ***P < 0.001. NS, no significance. One representative of three experiments.

Since Paneth cells residing in the small intestinal crypts of Lieberkühn secrete anti-microbial peptides, cytokines, and other trophic factors to maintain balance of the gut microbiota and kill pathogens such as *Shigella* spp., *Salmonella* spp., *Clostridium difficile, Escherichia coli* (*E. coli*) and *Citrobacter rodentium*, ^2,7,41^ we first used *Salmonella* Typhimurium (*S*.T) infection models ^42^ to investigate whether FABP4 can exert roles in controlling infection. After mice were infused using *S*.T (5 × 10^7^ colony forming unit (CFU)s) (Figure 2a), these FABP4^fl/fl^pvillin^CreT^ mice had slighter symposium, including longer gut length (Figure 2b), less inflammatory CD11b^+^Ly6G^+^cells and cytokines as compared to FABP4^fl/fl^ mice (Figure 2c, d), indicating that there are lighter inflammatory responses in FABP4^fl/fl^pvillin^CreT^ mice. Inflammation in the cecum and distal ileum was significantly slighter in FABP4^fl/fl^pvillin^CreT^ mice as compared to their control FABP4^fl/fl^ mice (Figure 2e). Less bacteria were also found in ileum, liver, lung and spleen in FABP4^fl/fl^pvillin^CreT^ mice (Figure 2f). While mice were challenged with *S*.T (2 × 10^2^ CFUs) (Figure 2g), FABP4^fl/fl^pvillin^CreT^ mice also showed lighter signs of progressive illness, including lighter ruffled fur, hunched posture and diarrhea with lower mortality and less lost body weight (Figure 2h, i). Lighter inflammations such as longer colon length, less inflammatory immune cells and bacterium burden in tissues were also observed in FABP4^fl/fl^pvillin^CreT^ mice (Figure S2). Meanwhile, we did also not found that FABP4 had any sex-specific effects on these symptoms. All of these indicate that FABP4^fl/fl^pvillin^CreT^ mice are highly resistance to *S*. T infection, implying higher bactericidal ability to pathogens in FABP4^fl/fl^pvillin^CreT^ mice. To further illustrate the role of FABP4 in controlling pathogens, we infused GFP-labelled *E. coli* (1 × 10^9^ CFUs) into mice, which are isolated from colitis tissues. ^43^ Data indeed showed stronger bactericidal ability to *E. coli* in FABP4^fl/fl^pvillin^CreT^ mice (Figure 2j-k). In addition, increased bactericidal ability against pathogens in FABP4^fl/fl^pvillin^CreT^ mice may also affect the composition of intestinal luminal microbiota,^7^ which are related to colitis and metabolic syndrome such as obesity. ^44,45^ We next used DSS-mediated colitis ^46^ and high-fat diet (HFD)-mediated obesity models to investigate these. FABP4^fl/fl^pvillin^CreT^ mice exhibited resistance to both DSS-mediated colitis and HFD-mediated obesity as compared to control FABP4^fl/fl^ mice (Figure S3 and Figure S4), implying that FABP4 also affects the composition of gut microbiota. Taken together, FABP4^fl/fl^pvillin^CreT^ mice have markedly increased bactericidal ability to pathogens.

### FABP4 in intestinal Paneth cells downregulates defensin expression and affects composition of gut microbiota

**Figure 3.**
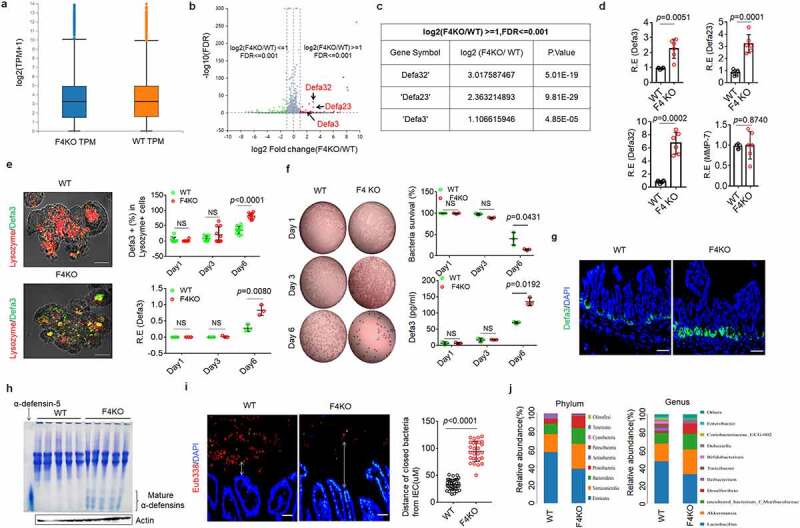
**FABP4 in intestinal Paneth cells downregulates defensin expression.** (a and b) RNA-seq of the gut organoids from FABP4^fl/fl^pvillin^CreT^ (F4KO) and FABP4^fl/fl^ (WT) mice (n=3). Box-plot (a) and volcano plot (b) of differential genes; α-defensin 32 (Defa 32), 23 (Defa 23) and 3 (Defa 3) (αdefensin 32/23 /3), which were labeled in red (b). (c) Gene expression of α-defensin 32/23 /3 in gut organoids of FABP4^fl/fl^pvillin^CreT^ (F4KO) and FABP4^fl/fl^ (WT) mice by RNA-seq analyses. (d) QRT-PCR of α-defensin 32/23/ 3 and MMP-7 in the gut organoid from FABP4^fl/fl^pvillin^CreT^ (F4KO) and FABP4^fl/fl^ (WT) mice (n=3). (e) Immunostaining of α-defensin 3 (Defa3) and lysozyme, and qRT-PCR of α-defensin 3 (Defa3) in the gut organoids of FABP4^fl/fl^pvillin^CreT^ (F4KO) and FABP4^fl/fl^ (WT) mice on day 1, day 3 and day 6. Data was shown only on day 6; Scale bar, 40 µm. (f) Killings of the supernatants on S. T, and ELISA of Defa3 of the supernatants in the gut organoids of FABP4^fl/fl^pvillin^CreT^ (F4KO) and FABP4^fl/fl^ (WT) mice at day1, day3 and day 6. CFUsonly on day 6 were shown. (g) Immunostaining of α-defensin 3 (Defa3) in the gut tissues of FABP4^fl/fl^pvillin^CreT^ (F4KO) and FABP4^fl/fl^ (WT) mice. DAPI (blue), nuclei. One representative (n=12). (h) AU-PAGE gel analysis of mature defensins of the ileum in FABP4^fl/fl^pvillin^CreT^ (F4KO) and FABP4^fl/fl^ (WT) mice (n=5). α-defensin-5, positive control. (i) Hybridization of fluorescence Eub338 probe in the ileum tissues of FABP4^fl/fl^pvillin CreT (F4KO) and FABP4^fl/fl^ (WT) mice. One representative (n=12). (j) 16S rRNA sequencing analyses of the pooled ileum contents from FABP4^fl/fl^pvillin ^CreT^ (F4KO) and FABP4^fl/fl^ (WT) mice (n=5). R.E, relative expression; Student’s t-testin d, e and f, mean ±SD; The Mann-Whitney U test in i. NS, no significance.

Defensins are critical bactericidal peptides in resisting against pathogens, especially *S*.T infection. ^2^ Thus, we next examined the expression of defensins in FABP4^fl/fl^pvillin^CreT^ mice. The RNA-Seq in the gut organoids on day 6 of FABP4^fl/fl^pvillin^CreT^ mice showed higher transcriptional levels of Defa (α-defensin) 32/23/3 than those of control FABP4^fl/fl^ mice (Figure 3a-c), indicating that FABP4 downregulates the expression of these defensins. Increased defensins in the gut organoid of FABP4^fl/fl^pvillin^CreT^ mice was further confirmed (Figure 3d). No differences were found in other bactericidal peptides such as β-defensin 1/ 2 /3, lyz1(lysozyme 1), reg3α, reg3γ, Saa1(serum amyoid A 1) and pla2g2a (recombinant phospholipase A2, group IIA) between FABP4^fl/fl^pvillin ^CreT^ mice and FABP4^fl/fl^ mice (Figure S5). Matrix metalloproteinase (MMP-7), which can mediate mature defensins, did also not change in both mice (Figure 3d). Defensin expression could be detected in gut organoids of mice after *in vitro* culture after 3 days (Figure 3e and Figure S6). The supernatants in the gut organoids of FABP4^fl/fl^pvillin^CreT^ mice also exhibited stronger bactericidal ability on *S*. T (Figure 3f). These results were further confirmed in the gut tissues of mice. Immunostaining and acid-urea polyacrylamide gel electrophoresis (AU-PAGE) showed that FABP4^fl/fl^pvillin ^CreT^ mice had higher levels of defensins in the intestinal tissues as compared to control FABP4^fl/fl^ mice (Figure 3g-h). Defensins possess a broad spectrum of antimicrobial activity and are bactericidal against Gram positive and negative bacteria, fungi, viruses, and unicellular parasites. ^4^ Markedly spatial segregations of microbiota and host in the intestine were observed in FABP4^fl/fl^pvillin^CreT^ mice (Figure 3i). Taken together, FABP4 in intestinal Paneth cells downregulates the expression of defensins.

The host defense peptide family of defensins also exerts noticeable effects on gut microbiota composition. ^47^ Indeed, the sequence analysis of the V3-V4 hypervariable regions of the 16S rRNA gene showed that microbiota composition was considerably alteration with a decrease in *Firmicutes* and an increase in *Bacteroides* in FABP4^fl/fl^pvillin^CreT^ mice as compared to FABP4^fl/fl^ mice (Figure 3j), which is opposite to HFD-mediated composition of gut microbiota in mice ^48–50^ and in patients with obesity.^51^ Not only in the phyla but also in the levels of genus and species, the proportions of bacteria in FABP4^fl/fl^pvillin^CreT^ mice were opposite to those in mice fed on HFD and in patient with obesity as compared to their controls (Figure S7). All of these indicate that FABP4 in Paneth cells can affect the composition of gut microbiota.

### FABP4-mediated downregulation of defensins in Paneth cells is through degrading PPARγ

**Figure 4.**
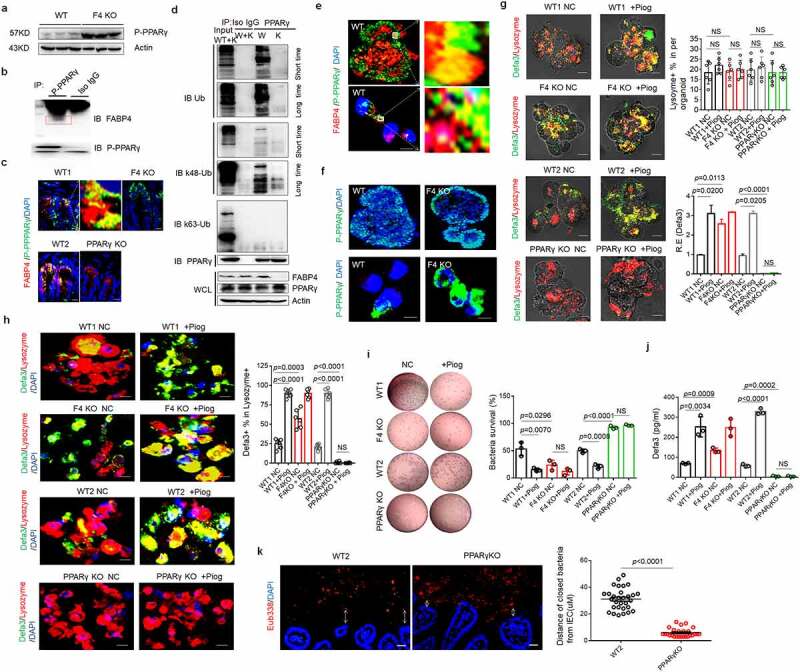
**FABP4 mediated downregulation of defensins in Paneth cells is through degrading PPARγ**. (a) Immunoblotting of P-PPARγ in the intestinal epithelial cells from FABP4^fl/fl^pvillin^CreT^ (F4KO) and FABP4^fl/fl^ (WT) mice (n = 3). Actin, a loading control. (b) Immunoblotting of FABP4 in the immune-precipitants with anti-P-PPARγ in the lyses of FABP4^fl/fl^pvillin ^CreT^ (F4KO) and FABP4^fl/fl^ (WT) mice. Iso IgG, isotypic antibody. (c) Immunostaining of P-PPARγ (green) and FABP4 (red) in FABP4^fl/fl^pvillin^CreT^ (F4KO) and FABP4^fl/fl^ (WT1) or WT2 and PPARγ KO mice. DAPI (blue), nucleus. Scale bar, 40 µm. (d) Immunoblotting of total Ub, k48-Ub, k63-Ub and PPARγ in the immune-precipitants with anti-PPARγ in the lyses of intestinal epithelial cells of FABP4^fl/fl^pvillin^CreT^ (K) and FABP4^fl/fl^ (W) mice. Iso IgG, isotypic antibody. (e) Immunostaining of FABP4 and P-PPARγ in the intestinal organoids of FABP4^fl/fl^pvillin^CreT^ (F4KO) and FABP4^fl/fl^ (WT) mice. Scale bar, 5 µm. (f) Immunostaining of P-PPARγ in the intestinal organoids of FABP4^fl/fl^pvillin^CreT^ (F4KO) and FABP4^fl/fl^ (WT) mice. Scale bar, 5 µm. (g) Immunostaining of α-defensin 3(Defa3) and lysozyme in the intestinal organoids of FABP4^fl/fl^pvillin^CreT^ (F4KO) and FABP4^fl/fl^ (WT1) mice, and WT2 and PPARγ KO mice with (Piog) or without (NC) pioglitazone. Scale bar, 40 µm. (h) Immunostaining of α-defensin 3(Defa3) and lysozyme in the cells of intestine organoids of FABP4^fl/fl^pvillin^CreT^ (F4KO) and FABP4^fl/fl^ (WT1) mice, and WT2 and PPARγ KO mice with (Piog) or without (NC) pioglitazone. Scale bar, 5 µm. (i) Killing on S.T in the supernatants of FABP4^fl/fl^pvillin^CreT^ (F4KO) and FABP4^fl/fl^ (WT1) mice, and WT2 and PPARγ KO mice with (Piog) or without (NC) pioglitazone. (j) ELISA of α-defensin 3(Defa3) in the supernatants of FABP4^fl/fl^pvillin^CreT^ (F4KO) and FABP4^fl/fl^ (WT1) mice, and WT2 and PPARγ KO mice with (Piog) or without (NC) pioglitazone. (k) Hybridization of fluorescent Eub338 in the ileum tissues of WT2 and PPARγ KO mice (n=6, 3 slides/ mouse). Scale bar, 40 µm. R. E, relative expression; Student’s t-test in g, h, i and j,mean ±SD. The Mann-Whitney U test in k. NC, untreated negative control. NS, no significance.

We next investigated mechanism(s) for FABP4 to downregulate defensins in Paneth cells. PPARγ is a major regulator of mucosal defenses. ^19,52^ The expression of defensins in *PPARγ* KO mice was remarkably decreased. ^19^ Transcription factor binding motif analyses revealed the enrichment for binding motifs of PPARγ on the promoter region of the defensins such as α-defensin 32/23/3 (https://biogrid-lasagna.engr.uconn.edu/lasagna search/). Others also found that FABP4 in adipose cells could trigger the ubiquitination and subsequent proteasomal degradation of PPARγ. ^53^ Thus, we next detected the levels of PPARγ expression in FABP4^fl/fl^pvillin^CreT^ mice. Higher levels of phosphorylated PPARγ (P-PPARγ) could be detected in the intestine epithelial cells of FABP4^fl/fl^pvillin^CreT^ mice as compared to FABP4^fl/fl^ mice (Figure 4a). Immunoprecipitation and immunostaining showed binding of FABP4 and P-PPARγ in gut epithelial cells (Figure 4b, c). K48 but not K63 ubiquitinated PPARγ could be detected by the immunoprecipitation with anti-PPARγ antibody, which was followed by immunoblotting with anti-ubiquitin antibody in FABP4^fl/fl^ mice not in FABP4^fl/fl^pvillin^CreT^ mice (Figure 4d). Since K48 but not K63 ubiquitination can prime degradation of proteins. ^54^ These results suggest that PPARγ can be degraded by FABP4 through K48 ubiquitination in Paneth cells. Binding of FABP4 with P-PPARγ was also confirmed using immunostaining *in vitro* cultured gut organoids of mice (Figure 4e). More P-PPARγs were observed and transported into nucleus in the *in vitro* cultured organoids of FABP4^fl/fl^pvillin^CreT^ mice than those of FABP4^fl/fl^ mice (Figure 4f). The increased defensins and bactericidal ability were also observed in FABP4^fl/fl^pvillin^CreT^ mice after exposure to pioglitazone, a specific agonist of PPARγ ^55^ (Figure 4g-j). All of these suggest that FABP4-mediated downregulation of defensins is through degrading PPARγ. In addition, the decreased defensins and bactericidal ability were also shown in *PPARγ* KO *in vitro* cultured organoids as compared to WT mice, especially after exposure to PPARγ stimulator pioglitazone (Figure 4g-j). There also had less spatial segregations of microbiota and host in the intestine in *PPARγ* KO mice (Figure 4k). We finally also investigated the effects of *PPARγ* KO on infection against *S*.T. After infusing *S*.T (5 × 10^7^ CFUs), *PPARγ* KO mice had markedly heavier symposiums, including stronger inflammatory in gut tissues, more bacteria in liver, lung and spleen (Figure S8a-f). These mice also showed heavier signs of progressive illness, with ruffled fur, hunched posture and diarrhea, and high mortality and lighter body weight while mice were challenged with lower numbers of *S*.T (2 × 10^2^ CFUs) (Figure S8g, h). Taken together, we demonstrate that FABP4-mediated downregulation of defensins is through degrading PPARγ in gut Paneth cells.

### HFD-mediated downregulation of defensins is through FABP4

**Figure 5.**
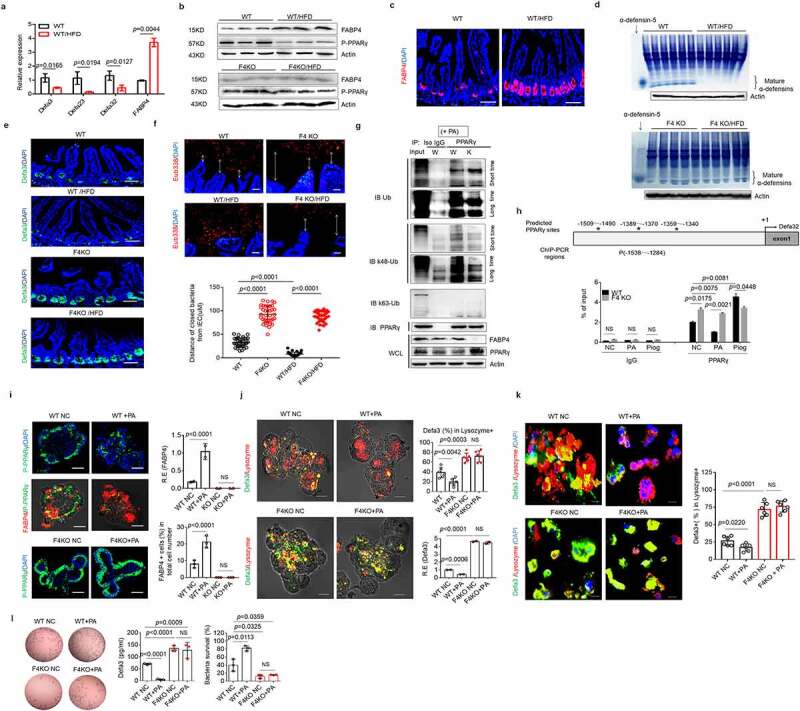
**HFD mediated downregulation of defensins is through FABP4.** (a)QRT-PCR of α-defensin 32/23/3 and FABP4 in the ileum tissues of mice with (WT/HFD) or without (WT) HFD for 4 weeks (Pooled samples, n=6). (b) Immunoblotting of FABP4 and P-PPARγ in the ileum tissues of FABP4^fl/fl^pvillin^CreT^ (F4KO) and FABP4^fl/fl^ (WT) mice with (WT/HFD or F4 KO/HFD) or without (WT or F4 KO) HFD for 4 weeks (Pooled samples, n=6). (c) Immunostaining of FABP4 (red) in the ileum tissues of FABP4^fl/fl^pvillin^CreT^ (F4KO) and FABP4fl/fl (WT) mice with(WT/HFD or F4 KO/HFD) or without (WT or F4 KO) HFD for 4 weeks. One representative (n=6). (d) AU-PAGE analysis of mature α-defensins in the ileum tissues of FABP4^fl/fl^pvillin^CreT^ (F4KO) and FABP4^fl/fl^ (WT) mice with (WT/HFD or F4 KO/HFD) or without (WT or F4 KO) HFD for 4 weeks. (e) Immunostaining of α-defensin 3 (Defa3) (green) in the ileum tissues of FABP4^fl/fl^pvillin^CreT^ (F4KO) and FABP4^fl/fl^ (WT) mice with (WT/HFD or F4 KO/HFD) or without (WT or F4 KO) HFD for 4 weeks. One representative (n=6). (f) Hybridyzation of fluoresence Eub338 probe in the ileum tissues of FABP4^fl/fl^pvillin^CreT^ (F4KO) and FABP4^fl/fl^ (WT) micewith (WT/HFD or F4 KO/HFD) or without (WT or F4 KO) HFD for 4 weeks. (g) Immunoprecipitation with anti-PPARγ or control (Iso IgG), immunoblotting of total Ub, K48-Ub, and K63-Ub in the ileum organoids in FABP4^fl/fl^pvillin^CreT^ (K) and FABP4^fl/fl^ (W) mice with PA. (h) Chip-PCR of PPARγ binding sits on the promoter region of α-defesin 32 with (PA or Piog) or without (NC) PA or pioglitazone. (i) Immunostaining of FABP4 and P-PPARγ in the intestinal organoids ofFABP4^fl/fl^pvillin^CreT^ (F4KO) and FABP4^fl/fl^ (WT) mice with (PA) or without (NC) PA.(j) Immunostaining of α-defensin 3 (Defa3) and lysozyme in the intestinal organoids of FABP4^fl/fl^pvillin^CreT^ (F4KO) and FABP4^fl/fl^ (WT) mice with (PA) or without (NC) PA. (k) Immunostaining of α-defensin 3(Defa3), and lysozyme in the cells of the intestinal organoids of FABP4^fl/fl^pvillin^CreT^ (F4KO) and FABP4^fl/fl^ (WT) mice with (PA) or without (NC) PA . Scale bar=5 µm. (l) Killing on S.T in the supernatants of the intestinal organoids of FABP4^fl/fl^pvillinCreT (F4KO) and FABP4^fl/fl^ (WT) mice with (PA) or without (NC) PA. NC in i-l, untreated negative control; Scale bar, 40  µm in c, e, f, i and j. R. E, relative expression; Student’s t-test, mean ±SD in a, h, i, j, k and l.The Mann-Whitney U test in f. NS, no significance.

The expression of defensins may be regulated by factors such as environmental factors. ^19,27^ We compared the expression of defensins in gut epithelial cells among new-born, 1–2 weeks, 4–5 weeks and 9 weeks old mice between FABP4^fl/fl^pvillin^CreT^ and FABP4^fl/fl^ mice. No markedly difference in new-born and 1–2 weeks old mice but significantly decreased expression in the defensins mainly including α-defensin 32/23/3 was found in the gut Paneth cells in 4–6 weeks old or older FABP4^fl/fl^ mice but not in FABP4^fl/fl^pvillin^CreT^ mice (Figure S9a-c), indicating that environmental factors such as that diet or gut microbiota affect the expression of defensins in FABP4^fl/fl^ mice. High-fat diet (HFD) is often associated with modification of the intestinal microbiota with higher proportion of *Firmicutes* ^56–58^ and also downregulate the expression of defensins. ^19,27,59^ We found that HFD could promote the expression of FABP4, reduce the levels of PPARγ and increase the distances between gut microbiota and gut epithelial cells in FABP4^fl/fl^ mice but not in FABP4^fl/fl^pvillin^CreT^ mice (Figure 5a-f and Figure S9d-f). All of these suggest that HFD-mediated downregulation of defensins is through FABP4.

Palmitic acid (PA) is a main component of HFD. ^60^ It could cause the expression of FABP4 in macrophages. ^61,62^ We found that PA could also cause the expression of FABP4, reduce defensins in gut epithelial cells and increase spatial segregations of microbiota and host in the intestine of mice (Figure S9g-i). Furthermore, markedly decreased defensins were detected in FABP4^fl/fl^ but not in FABP4^fl/fl^pvillin^CreT^ mice fed PA (Figure S10). PA also promoted PPARγ K48 ubiquitination in the gut epithelial cells of FABP4^fl/fl^ but not in FABP4^fl/fl^pvillin^CreT^ mice (Figure 5g). There had less accumulation of PPARγ on the promoter region of defensins after exposure to PA in FABP4^fl/fl^ mice, whereas similar accumulation of PPARγ on the promoter region of defensin 32 in both FABP4^fl/fl^ and FABP4^fl/fl^pvillin^CreT^ mice was found after pioglitazone (Figure 5h). *In vitro* cultured small intestine organoids also showed that PA could reduce PPARγ expression and P-PPARγ into nucleus in FABP4^fl/fl^ organoids but not in FABP4^fl/fl^pvillin^CreT^ organoids (Figure 5i). Immunostaining and qRT-PCR showed decreased expression of the defensins in the gut organoids of FABP4^fl/fl^ but not in FABP4^fl/fl^pvillin^CreT^ after exposure to PA (Figure 5j, k). The supernatants of *in vitro* cultured gut organoids of FABP4^fl/fl^ mice after exposure to PA also exhibited reduced bactericidal ability to *S*.T (Figure 5l). All of these suggest that PA-mediated downregulation of defensins is through FABP4. G-protein-coupled receptor (GPR)40, a receptor of long-chain fatty acid, which can be expressed by enteroendocrine I, K, and L cells, ^63^ was also expressed in the crypt cells (Figure S11a). Unlike to WT mice, increased expression of FABP4, reduced defensins and P-PPARγ were not found in *GPR40* KO mice (Figure S11b-e), suggesting that GPR40 play a critical role in PA-mediated downregulation of defensins. We also employed germ-free (GF) mice to further confirm the regulation of PA in FABP4, PPARγ and defensins. Infusion of PA but not omega-3 fatty acid caused the increased FABP4, the reduced P-PPARγ and defensins in GF mice (Figure S12). Taken together, all of these indicate that HFD can inhibit expression of defensins through upregulating FABP4.

### FABP4 in the Paneth cells of mouse colon downregulates defensin expression

**Figure 6.**
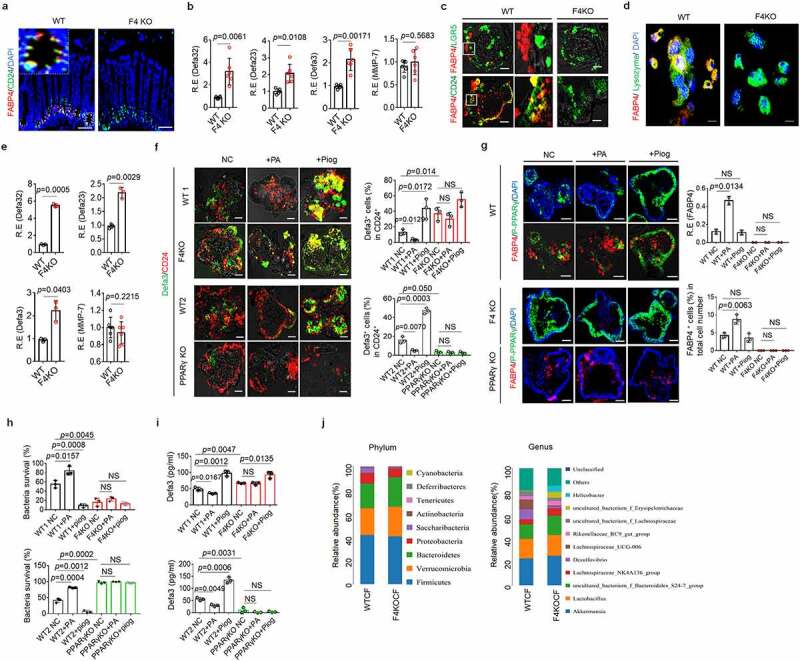
**FABP4 downregulates expression of defensins in mouse colon.** (a) Immunostaining of FABP4 and CD24 in the colon tissues of FABP4^fl/fl^pvillin^CreT^ (F4KO) and FABP4^fl/fl^ (WT) mice. One representative (n=6). (b) QRT-PCR of α-defensin32/23/3 and MMP-7 in the colon tissues of FABP4^fl/fl^pvillin^CreT^ (F4KO) and FABP4^fl/fl^ (WT) mice (n=6). (c) Immunostaining of FABP4, CD24 and LGR5 in the colon organoids of FABP4^fl/fl^pvillin^CreT^ (F4KO) and FABP4^fl/fl^ (WT) mice. (d) Immunostaining of FABP4 and CD24 in the cells of colon organoids of FABP4^fl/fl^pvillin^CreT^ (F4KO) and FABP4^fl/fl^ (WT) mice. Scale bar=5 µm. (e) QRT-PCR of α-defensin32/ 23/3 and MMP-7 in the colon organoids of FABP4^fl/fl^pvillin^CreT^ (F4KO) and FABP4^fl/fl^ (WT) mice. (f) Immunostaining of α-defensin 3 (Defa3) and CD24 in the colon organoids of FABP4^fl/fl^pvillin^CreT^ (F4KO) and FABP4^fl/fl^ (WT1)mice, and WT2 and PPARγ KO mice after exposure to PA or pioglitazone (Piog). (g) Immunostaining of FABP4 and P-PPARγ in the colon organoids of FABP4^fl/fl^pvillin^CreT^ (F4KO) and FABP4^fl/fl^ (WT1) mice, and WT2 and PPARγ KO mice after exposure to PA or pioglitazone (Piog). (h) Killing on S.T in the supernatants of colon organoids of FABP4^fl/fl^pvillin^CreT^ (F4KO) and FABP4^fl/fl^ (WT1)mice, and WT2 and PPARγ KO mice after exposure to PA or pioglitazone (Piog). (i) Elisa of α-defensin 3 (Defa3) in the supernatants of FABP4^fl/fl^pvillin^CreT^ (F4KO) and FABP4^fl/fl^ (WT1) mice, and WT2 and PPARγ KO mice after exposure to PA or pioglitazone (Piog). (j) 16S DNA analyses of the pooled colon contents in FABP4^fl/fl^pvillin^CreT^ (F4KOCF) and FABP4^fl/fl^ (WTCF) mice after exposure to HFD for 4 weeks (n-6). NC in f-i, untreated negative control; R. E, relative expression; Scale bar=40 µm in a, c, f and g; Student’s t-test, mean ±SD. NS, no significance.

Colon tissues possess Paneth-like cells. ^64,65^ These Paneth-like cells (CD24^+^cells) ^66^ in colon also expressed FABP4 (Figure 6a). Colonic epithelial cells can secret a large amount of host defense peptides against enteropathogenic bacteria such as *Shigella* spp., *Salmonella* spp., and *E. coli*. ^7^ Colonic epithelial cells from FABP4^fl/fl^pvillin^CreT^ mice could express more defensins as compared to the colonic epithelial cells from FABP4^fl/fl^ mice (Figure 6b). The FABP4 in colonic Paneth-like cells was further confirmed in *in vitro* cultured colonic organoids (Figure 6c, d). After exposure to PA, there had less defensins in the colon organoids of FABP4^fl/fl^ mice than those of FABP4^fl/fl^pvillin^CreT^ mice (Figure 6e, f). No changes were found in *PPARγ* KO mice after exposure to PA or pioglitazone (Figure 6f). Colon organoids in FABP4^fl/fl^ mice also expressed higher levels of FABP4 and less P-PPARγ after exposure to PA, whereas these did not happen in the colon organoids from FABP4^fl/fl^pvillin^CreT^ mice (Figure 6g). Unlike those in FABP4^fl/fl^ mice, PA did not affect the P-PPARγ to enter nuclei in FABP4^fl/fl^pvillin^CreT^ mice (Figure 6g). PA also decreased bactericidal ability to *S*.T in the colon organoids of FABP4^fl/fl^ but not in those of FABP4^fl/fl^pvillin^CreT^ mice (Figure 6h, i). However, pioglitazone promoted the bactericidal to *S*.T in the colon organoids of FABP4^fl/fl^ mice (Figure 6h, i). In addition, PA and pioglitazone did not affect bactericidal to *S*.T in *PPARγ* KO mice (Figure 6h). 16S rRNA analyses in the colon contents also exhibited difference in the composition of gut microbiota, typically increased ratio of *Bacteroidetes* and decreased *Firmicutes* in FABP4^fl/fl^pvillin^CreT^ mice after HFD (Figure 6j), further supporting the regulation of FABP4 in the defensins of colon epithelial cells. Taken together, FABP4 in the Paneth-like cells of colonic epithelial cells downregulates defensins through PPARγ.

### FABP4 in human Paneth cells downregulates defensin expression

**Figure 7.**
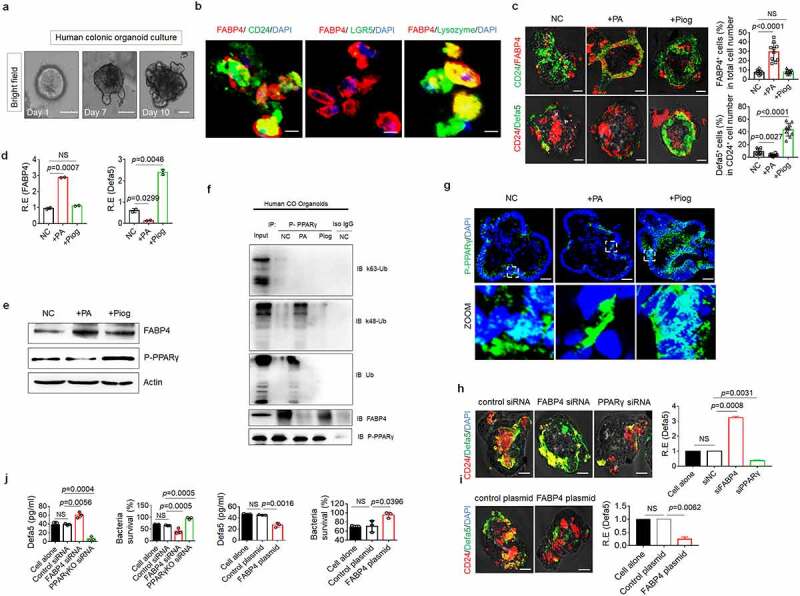
**FABP4 downregulates expression of the defensins in human colon.** (a) Bright fields of in vitro cultured human colonic organoids on day 1, day 7 and day 10. (b) Immunostaining of FABP4, CD24, LGR5 and lysozyme in the cells from human colonic organoids. Scale bar=5 µm. (c) Immunostaining of FABP4, CD24 and α-defensin 5 in human colonic organoids after exposure to PA or pioglitazone (Piog). (d) QRT-PCR of FABP4 and α-defensin 5 (Defa5) in human colonic organoids. (e) Immunosblotting of FABP4 and P-PPARγ in human colonic organoids after exposure to PA or pioglitazone (Piog). (f) K48-Ub in the human colonic organoids upon exposure to PA. Immunoprecipitation with anti-P-PPARγ or control (Iso IgG), immunoblotting of total Ub, K48-Ub and K63-Ub in the human colonic organoids upon exposure to PA. (g) Immunostaining of P-PPARγ in human colonic organoids after exposure to PA or pioglitazone (Piog). DAPI, blue. (h) Immunostaining of CD24 and α-defensin 5 (Defa5), and qRT-PCR of α-defensin 5 in FABP4 or PPARγ siRNA transfected human colonic organoids on day 10. Control siRNA, control siRNA transfected cells. (i) Immunostaining of CD24 and α-defensin 5 (Defa5), and qRT-PCR of α-defensin 5 in exogenous FABP4 (FABP4 plasmid) transfected human colonic organoids on day 10. Control plasmid, empty plasmid transfected cells. (j) ELISA of α-defensin 5 (Defa5) in the supernatants, and killing of the supernatants on the S. T in FABP4 and PPARγ siRNA or exogenous FABP4 transfected human colonic organoids on day 10. Control siRNA, control siRNA transfected cells; Control plasmid, empty plasmid transfected cells. NC in c-g, untreated negative control; R. E, relative expression; Scale bar=40 µm in a, c, g, h and i; Student’s t-test, mean ±SD. NS, no significance.

Finally, we investigated whether the regulation of FABP4 in the expression of defensin(s) also existed in human gut Paneth cells. Human colon CD24^+^ cells, which are Paneth-like cells ^67^ but not LGR5^+^ cells can express FABP4 (Figure S13). The expression of FABP4 was further confirmed in the CD24^+^ cells of *in vitro* cultured colonic organoids (Figure 7a, b). Most abundant intestinal host defense peptides in human are defensin 5(HD5) and defensin 6 (HD6), ^68^ which also have the enrichment binding motifs for the nuclear receptor PPARγ on the promoter region (https://biogrid-lasagna.engr.uconn.edu/lasagna_search/). Increased FABP4 and decreased defensin 5 were detected *in vitro* culture human gut organoids after exposure to PA (Figure 7c-e); Whereas only increased defensin 5 was found in those human gut organoids after exposure to pioglitazone (Figure 7c, d). Binding of FABP4 with PPARγ in *in vitro* cultured human colonic organoids was also detected using immunoblotting (Figure 7f). PA but not pioglitazone could promote PPARγ K48 ubiquitination in the human colonic organoids (Figure 7f). There was also less P-PPARγ in the nuclei of *in vitro* cultured organoids (Figure 7g) after exposure to PA not pioglitazone. Silencing FABP4 markedly upregulated the transcription levels of defensins and also promoted bactericidal ability to *S*.T, whereas exogenous FABP4 reduced defensin 5 expression and bactericidal to *S*.T (Figure 7h, i). In addition, silencing PPARγ also affected the production of defensins (Figure 7j). Thus our data suggest that FABP4 in the colon epithelial Paneth cells of human also controls the expression of defensins through degrading PPARγ.

**Figure 8.**
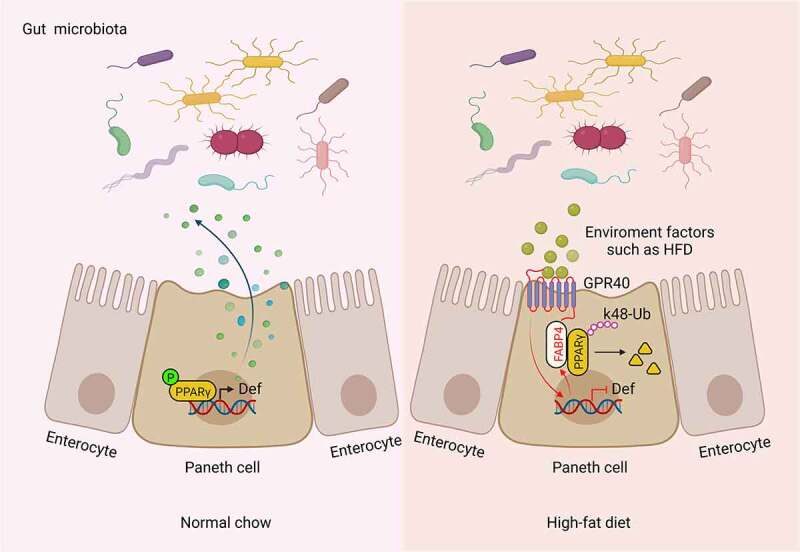
The mechanism for FABP4 exression in Paneth cells to regulate antimicrobial proteins.

## Discussion

Defensins possess a broad spectrum of antimicrobial activity, maintain the homeostasis of gut microbiota, and modulate numerous cellular responses, which are crucial for gut defenses. We here found that FABP4 expressed in the Paneth cells of small intestine and colon can downregulate defensins expression through degrading PPARγ by K48 ubiquitination (Figure 8). HFD may modulate host defense peptides expression ^27,69,70^ and lead to a downregulation of host defensins and PPARγ. ^19,27^ But, little is known how HFD regulates the expression of host defensins. We demonstrate that the expression of FABP4 in gut Paneth cells can be promoted by HFD, especially PA, a main component in HFD. Lower *Firmicutes*/*Bacteroidetes* ratio, which is opposite to that (higher *Firmicutes*/*Bacteroidetes* ratio) in mice fed HFD ^48–50^ was found in FABP4^fl/fl^pvillin^CreT^ mice. Importantly, FABP4-mediated downregulation of defensins in Paneth cells not only happens in mice but also in human. Since alternation of the gut microbiota composition is related to multiple diseases such as metabolic symposium, our results imply that FABP4 might be a target for therapy against these diseases. These findings will also offer basic information for how to regulate infection and maintain gut homeostasis by Paneth cells.

FABP4-mediated downregulation of defensins in Paneth cells is through degrading PPARγ. In adipose cells, FABP4 could also trigger the ubiquitination and subsequent proteasomal degradation of PPARγ, which consequently inhibit PPARγ-related functions. ^53^ PPARγ has been shown to be a major regulator of mucosal defenses.^19,52^ PPARγ activation is also required for maintenance of innate antimicrobial immunity in the colon. ^19^ PPARγ is a direct regulator of defensin expression in the human and mouse colons. ^19^

We demonstrate that HFD can promote the expression of FABP4, which can degrade PPARγ to reduce defensin expression. Defensins play a critical role in maintaining homeostasis of gut microbiota. ^7,9,10^ Indeed, HFD can change the composition of gut microbes. ^71–73^ Most studies have suggested that HFD raises the ratio of *Firmicutes* to *Bacteroidetes* and directly influences the assembly of an alternative microbiota. ^74,75^ The gut microbiota of obese animals and humans exhibits a higher *Firmicutes/Bacteroidetes* ratio compared with normal-weight individuals, which is proposed as an eventual biomarker. The higher *Firmicutes*/*Bacteroidetes* ratio is also frequently cited in the scientific literature as a hallmark of obesity.^48–50^ Especially, recent research found that HFD rather than obesity drives taxonomical and functional changes in the gut microbiota in mice. ^76^ In FABP4^fl/fl^Villin^CreT^ mice, the composition of gut microbiota could associate with a lower *Firmicutes/Bacteroides* ratio, which are opposite to that in mice fed a HFD as compared to mice fed on a normal diets. ^48–50^ Thus, that HFD-induced FABP4 in Paneth cells downregulates defensin 32/23/3 through degrading PPARγ might be a main reason for the alteration of gut microbiota composition in mice fed HFD.

A major function of enteric defensins is the protection from intestinal pathogens. ^77^ However, FABP4^fl/fl^pvillin^CreT^ mice, which express higher levels of defensins, show an increased frequency of Phylum *Proteobacteria*. This may be that gut microbiota such as some bacteria, which can inhibit *Proteobacteria*, are killed by increased defensins in FABP4^fl/fl^pvillin^CreT^ mice. There exist differences in the sensitivity of bacteria to the defensins. ^78,79^ Notably, the pathogenic bacteria such as *Escherichia* and *Klebsiella* species in Phylum *Proteobacteria*, did not significantly increase in genus level, suggesting that the gut microbiota in FABP4^fl/fl^pvillin^CreT^ mice promoted increases of other some species in *Proteobacteria*. The phylum *Proteobacteria* is the most unstable over time among the four main phyla (*Firmicutes, Bacteroidetes, Proteobacteria*, and *Actinobacteria*) in the gut microbiota. ^80^

In addition, lower levels of human defensins have been described in ileal Crohn’s disease. ^15,81–83^ In two German cohorts patients with ileal Crohn’s disease had reduced levels of HD5 and HD6. ^15^ Another cohort in Australia also had decreased levels of HD5. ^82^ Recent reports exhibit high expression of FABP4 in patient with colorectal cancer by immunohistochemistry and western blot. ^84^ The regulation of FABP4 in the defensins in Paneth cells might also offer a target for interfering these diseases.

## Materials and methods

### Reagents and oligoes

Reagents and oligoes used in this study were listed in Table S1.

### Mice, human tissues and bacterium strains

Four-to six-week-old male or female C57BL/6 mice were obtained from Nanjing Animal Center, Nanjing, China. *PPARγ* KO mice in B6 background were from Prof. Cao, Nanjing University. *GPR40* KO mice in B6 background were from BRL Medicine Inc. Shanghai, China. All mice were bred and kept under specific pathogen-free (SPF) condition in Animal Center of Nankai University. C57BL/6 germ-free (GF) mice were generated by Beijing Animal Center. All experiments in GF mice were performed in Institute of Laboratory Animal Science, Beijing, China. Experiments were carried out using age- and gender- matched mice. All procedures were conducted according to the Institutional Animal Care and Use Committee of the Model Animal Research Center. Animal experiments were approved by the Institute’s Animal Ethics Committee of Nankai University. All experimental variables such as husbandry, parental genotypes, and environmental influences were carefully controlled.

FAPB4^fl/fl^pvillin-creT mice (Gut epithelial cell FABP4 conditional knockout mice) and control FABP4^fl/fl^ mice were prepared via CRISPR/Cas9 system by Gempharmatech, Nanjing, China according to previously reported methods by us with modifications. ^85^ Firstly, two gRNAs-targeting the Fabp4 gene were respectively constructed and transcribed *in vitro*. The donor vector with the Loxp fragment was also designed and constructed *in vitro*. Then Cas9, gRNA and donor were co-injected into zygotes. The F0 and F1 mice were identified using a standard PCR-based genotyping procedure with following primers, FABP4 -B5S8/B3S2-D-5in–F1, 5’-GGCCTAAGAATGTGTGATTA-3’ and ZMK1R3, 5’-AAGGGTTATTGAATATGATCGGA-3’, yields a 1055bp products; FABP4 – B5S8/B3S2-D-2nd-TF2, 5’-ACAGTAGTGCATGTGGAGTA-3’ and FABP4 – B5S8/B3S2-D-2nd-TR2, 5’-AAGTTAGATGGCAGACACAT-3’, yields a 126bp product from wild-type allele and a 224bp product from floxed allele. For breeding, Fabp4 flox mice were mated with pvillin-cre to obtain conditional knockout heterozygous mice, and the genotyping was fl/wt, creT. Fl/fl creT, and Fl/fl creW mice were obtained by fl/wt, creT mating with fl/fl.

Human colonic tissues were obtained from patients at the Tianjin People’s Hospital (TPH). The TPH institutional review board committee and Ethics *Committee* on the use of humans as experimental subjects approved the protocols.

*Salmonella* Typhimurium (*S*. T, ATCC14028) was cultured in MacConkey Agar (Oxoid) liquid medium at 37°C with shaking at 200 rpm. GFP-labelled *E. coli* 0160 were from colitis tissues,^43^ and cultured in LB medium at 37°C with shaking at 200 rpm.

### Mouse models

For chronic *S*. T infection, FBAP4^fl/fl^ and FBAP4^fl/fl^pvillin-cre^T^ mice were first fasted for 4 h, and then mice were orally gavaged streptomycin (20 mg/Kg) and refed for 20 h. Then mice were fasted again for 4 h before each mouse was orally gavaged with *S*. T (2 × 10^2^ CFUs) in a total volume of 300 μl sterile PBS at day 0. After infection for 6 days, the bacterial in the ileum, spleen, liver and lung tissues from individual mouse was counted in MacConkey Agar (Oxoid) plates. Mice survival rate and body weight were monitored. For acute *S*. T infection model, the infection protocol was consisted with chronic *S*. T infection model except each mouse was orally gavaged with *S*. T (5 × 10^7^ CFUs) in a total volume of 300-μl sterile PBS at day 0. After infection for 24 h, mice were euthanized for further analysis.

For infection of GFP-labelled *E. coli*, previously reported method by us was used with modification. ^85^ Briefly, mice were first treated with pan-antibiotics vancomycine (V, 0.5 g/L, Sigma), ampicillin (A, 1 g/L, Sigma), neomycin sulfate (N, 1 g/L, Sigma), and metronidazole (M, 1 g/L, Sigma) via the drinking water for one week, and then orally administered 200 μl of GFP-labelled *E. coli* (1 × 10^9^ CFUs).

For dextran sodium sulfate (DSS) induced colitis, DSS induced colitis was performed according to our previously reported method.^86^

For high-fat diet model, previously reported method by us was used with modification.^85^ Briefly, 6–8 weeks old male mice were fed using high-fat diet (D12492, protein (26.2%), carbohydrate(26.3%) and fat (34.9%)) and control diet (D12450B), which was from Research Diets, Inc.(NJ, USA) according to reported protocol.^87^ Mice were sacrificed and representative analyses were performed at indicated time. For other oral administration, mice were orally gavaged with 300 mg/kg of palmitic acid (MCE, HYN0830) solution, or 100 mg/kg of linoleic acid solution (MCE, HY-N0729), 300 mg/kg of eicosapentaenoic acid solution (MCE, HY-B0660) or 10 mg/kg pioglitazone (MCE, HY-13956) solution at day 0. All mice were allowed free access to water and chow during the experiments. After orally administrated for 2 days, mice were sacrificed and representative intestinal or colonic tissues were harvested for detail analyses. Body weight changes, disease activity index (DAI) and histological score were assessed according previously reported methods by us. ^30^

### In vitro cultured organoids

Gut organoids cultures were basically according to reported methods. ^39^ Briefly, crypts were incubated for 15 min at room temperature (RT) in gentle cell dissociation reagent (Stemell technologies), then crypts were passed through 70 μm cell strainer (biosharp, BS-70-XBS) and collected by centrifugation at 100 × g for 5 min. The crypts were embedded with Matrigel (Corning) and plated. Carefully transfer the plate to a 37°C incubator. Incubate at 37°C for 10 min to allow domes to polymerization. After polymerization of matrigel, the crypts were added specifically IntestiCult^TM^ medium (Stemcell technologies) and placed plate in incubate at 37°C and 5% carbon dioxide. The culture medium should be fully exchanged 3 times per week. For mouse colonic organoid culture, colonic tissues were separated and crypts were incubated for 25 min at RT in gentle cell dissociation reagent (Stemcell technologies), then the detail operations were same to the intestinal organoid culture. For human colonic organoid culture, freshly colonic tissues were washed with ice-cold PBS to remove adipose tissues and blood, then incubated at gentle cell dissociation reagent for 25 min at RT. Crypts were enriched by centrifugation and embedded with matrigel and plated. After matrigel fully polymerization at 37°C, 750 μl of complete human intestiCult organoid growth medium (Stemcell technologies) supplemented with 10 μM final concentration Y-27632 (MCE) for primary culture was added, and incubated at 37°C and 5% CO_2._ Every 2 days, a full medium change with complete human intestiCult organoid growth medium (Y-27632 is not required) was performed.

### Ex vivo stimulation

For stimulation on *in vitro* cultured organoids, gut organoids were first cultured to day 5 or colonic organoids were cultured to day 9. Then the organoids were stimulated with 30 μM palmitic acid or 0.9330 μM pioglitazone for 24 h. Then the organoids were collected for further analyses.

### Immuno-staining

For *in vitro* cultured organoid staining, the organoids were incubated with dispase (1 mg/mL) (Corning Life Sciences) for 30 min at 37°C to partially dissolve the matrigel. Organoids were collected and gently spun down at 100 × g for 3 min. Organoids were fixed with 1 ml 4% paraformaldehyde at least 1 h at room temperature (RT) or 4°C overnight. Organoids were washed 3 times with 1× PBS, gently spun down at 100 × g for 3 min, or allowed to settle to the bottom of the tube by gravity after each wash. Organoids were blocked with 1× blocking buffer (1× PBS + 5%BSA + 0.3% Triton X-100) for 1 hr at RT, and incubated with primary antibodies in antibody dilution buffer (1× PBS+1% BSA+0.3% Triton-X) overnight at 4°C. After washing 3 times with PBS for 10 min each wash. Organoids were incubated with secondary antibodies with 1× secondary antibody buffer (1× PBS + 0.3% Triton X-100) for 2 h at RT in the dark. Organoids were washed 3 times with 1× PBS for 10 min each wash. Nucleus was stained with DAPI. Finally, organoids were pipetted with a barrier pipette tip onto raised chamber slides, and were observed using confocal microscope. For whole organoid image statistic analyses, associated fluorescence intensity or positive cells counter were quantified by ImageJ software.

For gut immune staining, previously reported methods by us was used.^30^ Briefly, mice intestinal and colonic tissues were fixed in 4% (w/v) paraformaldehyde buffered saline, then and embedded in Paraffin. 5-μm-thick sections were prepared and stained with primary antibodies in 5% normal goat serum blocking buffer overnight at 4°C. After washing, sections were incubated with fluorescent labeled secondary antibody for 2 h at room temperature. Observations were performed with a Zeiss LSM 700 confocal microscope.

### Gut microbiota analysis

For gut microbiota analyses, previously reported protocols by us ^85^ were used. Briefly, gut microbiota was analyzed by Majorbio Biotechnology Company (Shanghai, China) using primers that target to the V3-V4 regions of 16S rRNA. Using sample-genus and sample-OTU count matrices, the samples were clustered at genus and OTU levels. For each clustering, we used Morisita-Horn dissimilarity to compute a sample distance matrix from the initial count matrix. Using Ward’s minimum variance method, the distance matrix was subsequently used to generate a hierarchical clustering. The Wilcoxon Rank Sum test was used to identify OTUs that had differential abundance in the different groups.

### Acid/urea-polyacrylamide gel electrophoresis (AU-PAGE gel)

For mature a-defensin detection, Acid/Urea-Polyacrylamide Gel Electrophoreses were performed. Briefly, the ileums were opened longitudinally and chopped into around 5 mm pieces. After washed with ice-cold PBS for three times, the tissue fragments were shaken in 5 mM EDTA at 4°C for 40 min. After the intestinal fragments by gravity to the bottom of the tube, the solution was filtered with a strainer (70 mm) to enrich for crypts. The crypts were divided into two parts, and one part was lysed with proteins lysing buffer to determine total protein amounts by SDS-PAGE for β-actin concentration. For whole proteins extraction, tissues were weighted and cut for small pieces, then RIPA lysis buffer was added and shaken over for 30 min on ice, centrifuged at 12000 rpm for 15 min and transferred the supernatants into a new EP tube and boiled 10 min at 100°C. Another part crypts were solubilized in 500 μl of AU-PAGE loading solution (10 ml loading buffer including 1.8 g Urea, 1.2 ml acetic acid, 0.005 g methyl green, and 400 μl TEMED) and denatured at 100°C for 10 min for denaturation. Equal amounts of proteins were supplied on a 12.5% AU-PAGE gel for 2.5 h in 2% acetic acid running buffer.

### Immunoprecipitation and immunoblotting

For immunoprecipitation and immunoblotting, the epithelial cells of mouse intestines or human colon organoids were collected, and cells then were lysed with non-denatured cell lysis buffer. The part of the whole cell lyses was prepared for input control with 5× loading buffer boiling 10 min at 100^o^C. The rest of cell lyses were incubated with anti-P-PPARγ, or isotypic antibodies for 4 h at 4°C, then added protein A/G magnetic beads into incubation buffer overnight at 4°C. Followed by washing with lyses washing buffer for 3 times (10 min each time), magnetic beads precipitate was denatured using 1× loading buffer at 100°C for 10 min. Samples then subjected to SDS-PAGE gel. The proteins on the gel were transferred to PVDF membranes and were incubated with anti-P-PPARγ, FABP4, Ub, K48-Ub, or K63-Ub or Actin overnight at 4°C. Secondary HRP-conjugated antibodies were incubated for 1 h at RT. After washed by washing buffer for 3 times, the membranes were detected using an enhanced chemiluminescence assay with Lumi-Glo reagents (Millipore).

### Chromatin immunoprecipitation (ChIP)-PCR

Chromatin immunoprecipitation (ChIP)-PCR was performed using EZ-ChIP™ Chromatin Immunoprecipitation Kit (Millipore) according to the our previously reported methods. ^88^ Briefly, the cells were crosslinked with 1% paraformaldehyde and incubated with rotation at room temperature. Crosslinking was stopped after 10 min with glycine to a final concentration of 0.125 M and incubated 5 min further with rotation. Cells were washed with ice cold PBS (containing 1% PMSF) 3 times and immediately resuspended in SDS lysis buffer (containing 1% PMSF). Cell lysates were sonicated for 40 cycles of 30 s ON and 30 s OFF in 10 cycle increments using a Biorupter (Diadenode) on ice. After pelleting debris, protein G agarose was added and incubated for 1 hour at 4°C with rotation for preclearing. For immunoprecipitation, precleared cell lysate was incubated with the indicated antibodies overnight with the rotation at 4°C and protein G agarose was added for the final 2 h of incubation. Beads were washed with low salt, high salt, LiCl wash buffer and chromatin immunocomplex was eluted using elution buffer through incubating at room temperature for 15 min. Reverse crosslinks of protein/DNA complexes to free DNA were realized through adding 5 M NaCl and incubating at 65°C overnight. qPCR was performed on DNA purified after treatment with RNase (30 min, 37°C) and proteinase K (2 h, 55°C) after reversal of crosslinks.

### Metabolism experiments

For glucose and insulin tolerance, after fasting for 5 h, baseline blood glucose levels were measured using a Nova Max Plus GlucoseMeter. Mice were then ip injected with 2 g glucose per kg body weight in sterile PBS or with 0. 5 U insulin (Sigma, St. Louis, Missouri) per kg body weight, and blood glucose levels were measured at different times.

### RNA-Seq analysis

Expression of genes in the *in vitro* cultured gut organoids of FABP4^fl/fl^pvillin^CreT^ mice and control FABP4^fl/fl^ mice was analyzed using RNA-seq by BGI, Wuhan, China according to our previously reported method. ^89^

### Fluorescence in situ hybridization (FISH)

For bacteria localization at the surface of the intestinal mucosa, FISH was performed according to previously reported method by us. ^90^

### Cell isolation and flow cytometry

For surface and intracellular staining of lamina propria (LP) lymphocytes, previously reported methods by us ^85^ were used.

### Quantitative reverse transcriptase-polymerase chain reaction analysis (qRT-PCR)

qRT-PCR was performed according to previously reported methods by us. ^30^ The primers used for qRT-PCR were shown in Table S1.

### Enzyme linked immunosorbent assay (ELISA)

For mouse Defa3 and human Defa5 ELISA measurement, the supernatants from *in vitro* cultured organoids were collected, and then assay were carried out according to the manufacturer’s instructions (Cloud-clone).

### Statistical analysis

Statistical analyses, including two-tailed Student t test, ONE-way ANOVA Bonferroni’s Multiple Comparison Test, the Mann–Whitney U test, Kaplan and Meier method, and Wilcoxon’s test were performed by GraphPad Prism 7 software (GraphPad Software). A 95% confidence interval was considered significant and was defined as *, P < 0.05; **, P < 0.01; ***, P < 0.001.

## Supplementary Material

Supplemental Material

## Data Availability

All relevant data are available in the Source Data or Extended Data Information of the manuscript. RNA-Seq GEO accession number for the gut organoids on day 6 of FABP4^fl/fl^pvillin^CreT^ mice and control FABP4^fl/fl^ mice: https://www.ncbi.nlm.nih.gov/geo/query/acc.cgi?acc=GSE200967; GEO accession number for 16S DNA analyses in the ileum and colon contents of FABP4^fl/fl^pvillin^CreT^ and FABP4^fl/fl^ mice: https://www.ncbi.nlm.nih.gov/Traces/study/?acc=PRJNA827574.

## Abbreviations

FABP4: Fatty acid binding protein 4; *S*. T: *Salmonella* Typhimurium; HFD: High-fat diet; Defa: α-defensin; HD5: Human α-defensin 5; HD6: Human α-defensin 6; F/B: Firmicutes/Bacteroidetes; SFB: Segmental filamentous bacteria; AMPs: Antimicrobial peptides; PPARγ: Peroxisome proliferator-activated receptor γ; P-PPAR: Phosphorylated PPAR; Dhx15: DEAD-box helicase 15; EGF: Epidermal growth factor; ENR: Noggin and R-spondin 1; CFU: Colony forming unit; Lyz1: Lysozyme 1; Saa1: Serum amyoid A 1; Pla2g2a: Phospholipase A2, group IIA; MMP-7: Matrix metalloproteinase; AU-PAGE: Acid-urea polyacrylamide gel electrophoresis; PA: Palmitic acid; GPR40: G-protein-coupled receptor; GF: Germ-free; EGF: Epidermal growth factor; LP: Lamina propria; KO: Knock out; WT: Wild-type.
