# Supplementary material for: FABP4 in Paneth cells regulates antimicrobial protein expression to reprogram gut microbiota
Source: Gut Microbes. 2022 Oct 31;14(1):2139978. doi: 10.1080/19490976.2022.2139978 (PMC9635462; doi:10.1080/19490976.2022.2139978)
Supplement: Supplemental Material [file KGMI_A_2139978_SM6654.docx]

**Supplementary data**

**FABP4 in Paneth cells regulates antimicrobial protein expression to reprogram gut microbiota**

Xiaomin Su^a*^, Mengli Jin^a,b,c*^, Chen Xu^d*^, Yunhuan Gao^a,b,c^, Yazheng Yang^a,b,c^,

Houbao Qi^a,b,c^, Qianjing Zhang^a,b,c^, Xiaorong Yang^a,b,c^, Wang Ya^a^, Yuan Zhang ^a,b,c^ & Rongcun Yang^a,b,c^


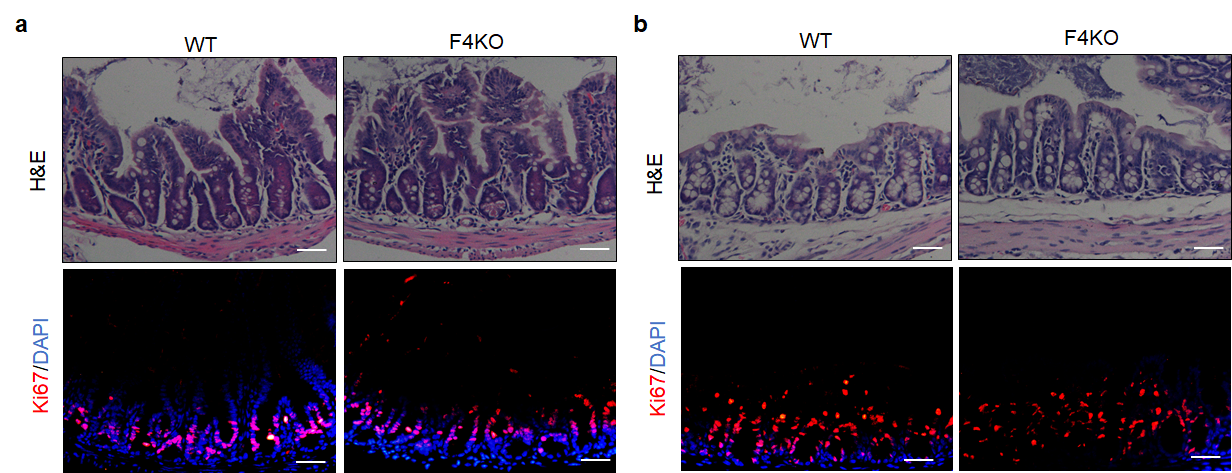


**Figure S1. FABP4 in Paneth cells does not affect the development of gut epithelial cells.** (a) H/E staining (upper) and immunostaining (lower) of Ki67 in the ileum of FABP4^fl/fl^pvillin^CreT^ (F4KO) and FABP4^fl/fl^ (WT) mice. (b) H/E staining (upper) and immunostaining (lower) of Ki67 in the colon of FABP4^fl/fl^pvillin^CreT^ (F4KO) and FABP4^fl/fl^ (WT) mice. One representative (n=8). Scale bar, 40 µm.


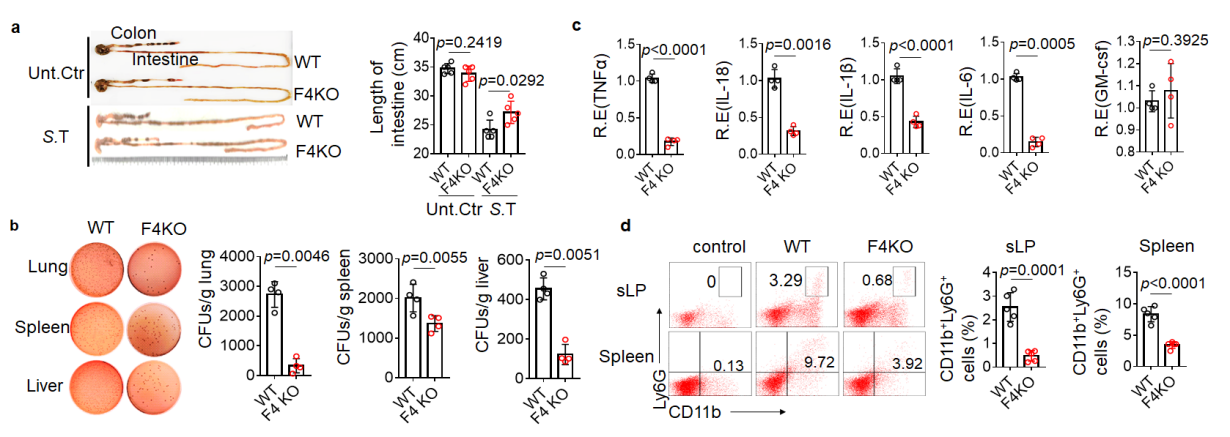


**Figure S2. FABP4^fl/fl^pvillin^CreT^ mice have markedly resistance against *S.*T infection.** (a) Length of the colons and intestines of FABP4^fl/fl^pvillin^CreT^ (F4KO) and FABP4^fl/fl^ (WT) mice with (*S.*T) or without (Unt. Ctr) chronic infection of *S.*T (2×10^2^ CFUs/mouse). (b) CFUs of *S.*T in the lung, spleen and liver of FABP4^fl/fl^pvillin^CreT^ (F4KO) and FABP4^fl/fl^ (WT) mice with chronic infection of *S.*T. The lung, spleen and liver tissues of mice were harvested and weighted, equal weight tissues from different groups were homogenized in equal of amount of PBS. Homogenates were serially diluted and plated on Salmonella chromogenic agar to quantify CFUs of *S.* typhimurium*.* (c)QRT-PCR of TNFα, IL-18, IL-6, IL-1β and GM-csf in the ileum of FABP4^fl/fl^pvillin^CreT^ (F4KO) and FABP4^fl/fl^ (WT) mice with chronic infection of *S.*T.

(d) Flow cytometry of CD11b^+^Ly6G^+^ cells in the ileum of FABP4^fl/fl^pvillin^CreT^ (F4KO) and FABP4^fl/fl^ (WT) mice with *S.*T chronic infection. Control, Ly6G isotypic control. Student’s t-test in per Panel, mean ±SD; R. E, relative expression; A representative of three independent experiments.


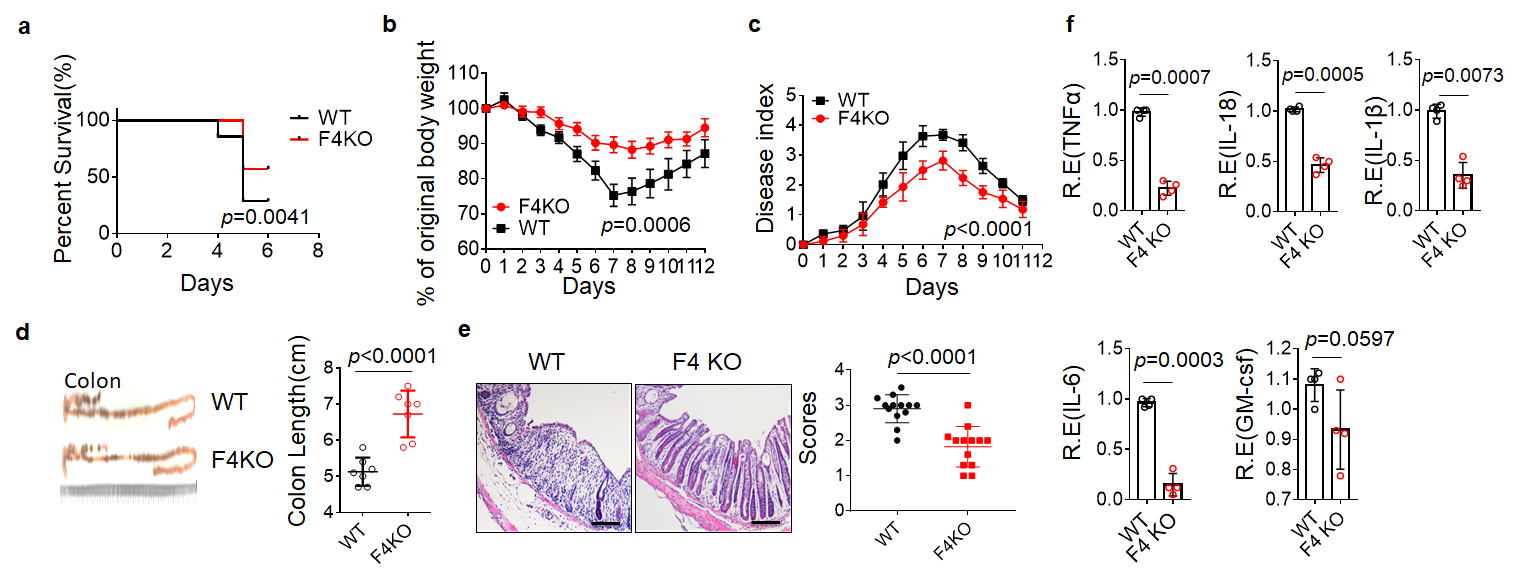


**Figure S3. FABP4^fl/fl^pvillin^CreT^ mice are more resistance to DSS-mediated colitis.**

(a) Survival rate of FABP4^fl/fl^pvillin^CreT^ (F4KO) and FABP4^fl/fl^ (WT) mice after exposure to DSS (2.5% DSS) for 8 days. n=12. (b) Body weight curve of FABP4^fl/fl^pvillin^CreT^ (F4KO) and FABP4^fl/fl^ (WT) mice after exposure to DSS (2.5% DSS). n=12. (c) Disease indexes of FABP4^fl/fl^pvillin^CreT^ (F4KO) and FABP4^fl/fl^ (WT) mice after exposure to DSS (2.5% DSS). n=12. (d) Colon length of FABP4^fl/fl^pvillin^CreT^ (F4KO) and FABP4^fl/fl^ (WT) mice after exposure to DSS (2.5% DSS). (e) H&E staining of colon tissues of FABP4^fl/fl^pvillin^CreT^ (F4KO) and FABP4^fl/fl^ (WT) mice after exposure to DSS (2.5% DSS). Scale bar=40 μm. (f) QRT-PCR of TNFa, IL-18, IL-1β, IL-6 and GM-csf in colonic tissues of FABP4^fl/fl^pvillin^CreT^ (F4KO) and FABP4^fl/fl^ (WT) mice after exposure to DSS (2.5% DSS).

Student’s t-test in panel d, e and f, mean ±SD; Analysis of variance test in b and c; Wilcoxon's test in a. Scale bar= 40 um. Data are a representative of three independent experiments; R. E, relative expression.


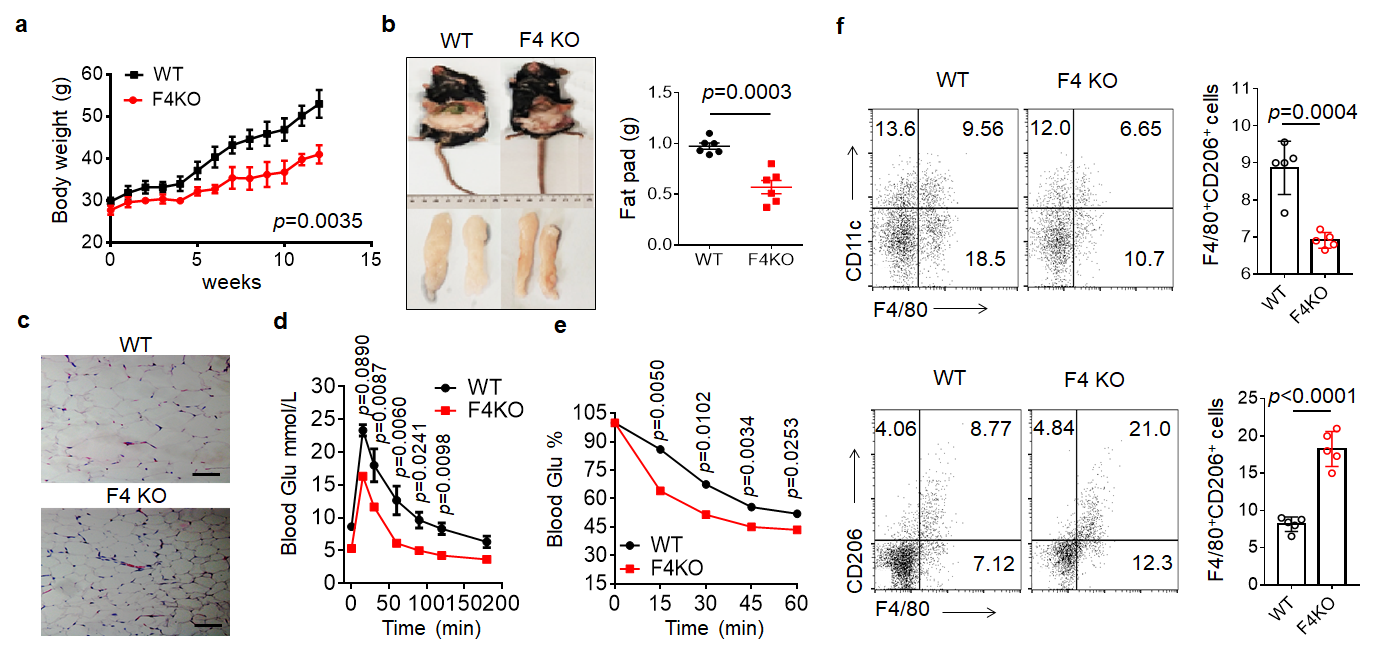


**Figure S4. FABP4^fl/fl^pvillin^CreT^ mice are resistance to HFD-mediated obesity.**

(a) Body weights of FABP4^fl/fl^pvillin^CreT^ (F4KO) and FABP4^fl/fl^ (WT) mice after giving HFD. n=12. (b) Adipose fatpad of FABP4^fl/fl^pvillin^CreT^ (F4KO) and FABP4^fl/fl^ (WT) mice after giving HFD for 12 weeks. One representative from 12 mice.

(c) H/E staining of adipose tissues FABP4^fl/fl^pvillin^CreT^ (F4KO) and FABP4^fl/fl^ (WT) mice after giving HFD for 12 weeks. One representative from 12 mice. Scale bar=40 µm. (d) Glucose tolerance in FABP4^fl/fl^pvillin^CreT^ (F4KO) and *FABP4^fl/fl^* (WT) mice. n=8. (e) Insulin tolerance in FABP4^fl/fl^pvillin^CreT^ (F4KO) and FABP4^fl/fl^ (WT) mice. n=8. (f) Flow cytometry of F4/80^+^CD11c^+^ inflammatory macrophages and F4/80^+^CD206^+^ immune suppressive macrophages in the adipose tissues of FABP4^fl/fl^pvillin^CreT^ (F4KO) and FABP4^fl/fl^ (WT) mice fed HFD for 12 weeks.

Student’s t-test in b and f, mean±SD；Analysis of variance test in a, d and e; A representative of three independent experiments; R. E, relative expression.


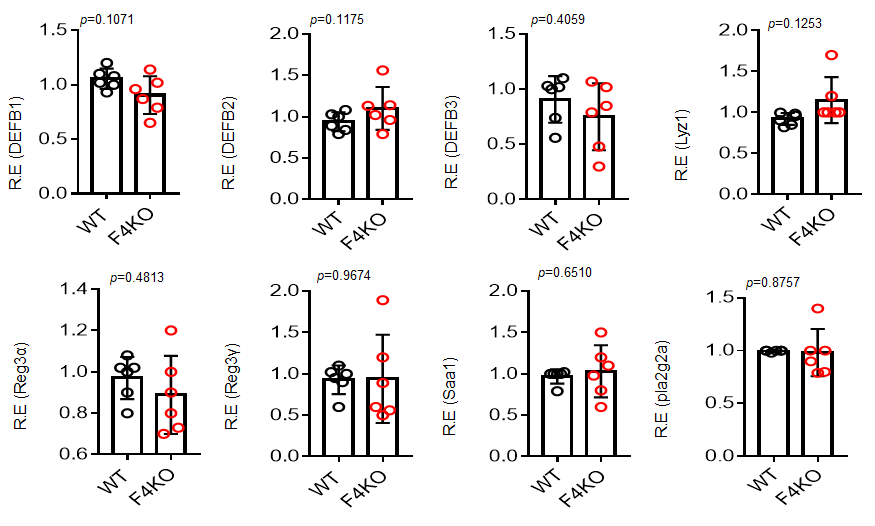


**Figure S5.** No difference in the expression of β-defensin 1/2/3 (DEFB1,B2 and B3), lyz1, reg3α(Reg3α), reg3γ(Reg3γ), Saa1 and pla2g2a in the gut organoid of FABP4^fl/fl^pvillin^CreT^ (F4KO) and FABP4^fl/fl^ (WT) mice. Student’s t-test, mean ± SD. A representative of three independent experiments; R. E, relative expression.


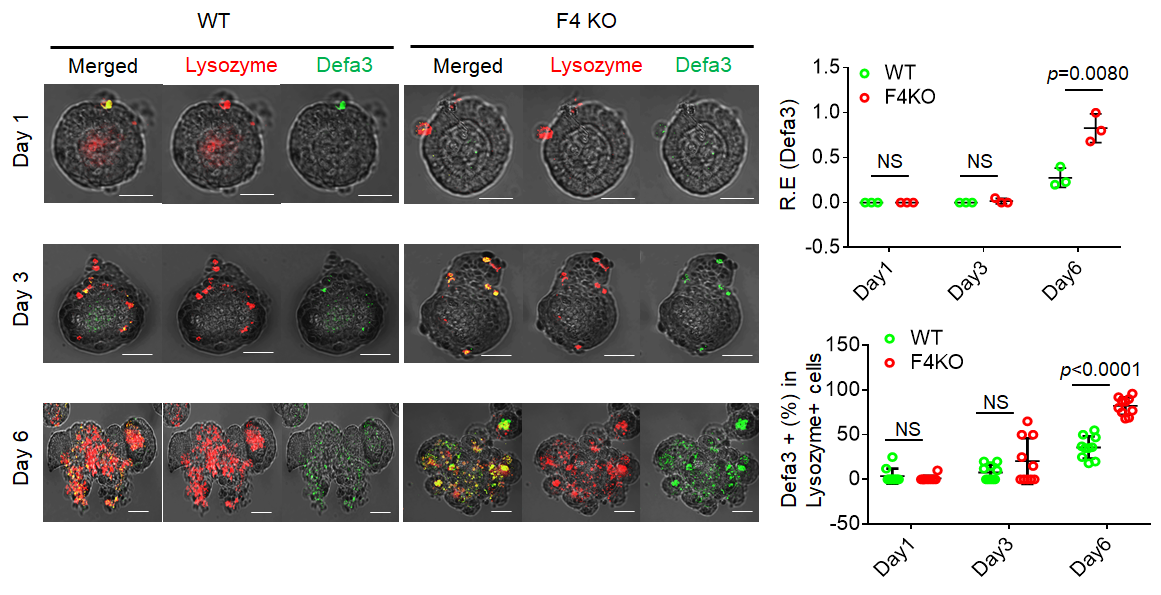


**Figure S6. *In vitro* gut organoids express defensins at different times.**  Immunostaining of α-defensin 3 (Defa3) and lysozyme, and qRT-PCR of Defa3 in *in vitro* cultured gut organoids of FABP4^fl/fl^pvillin^CreT^ (F4KO) and FABP4^fl/fl^ (WT) mice on day 1, day 3 and day 6. Student’s t-test, mean ± SD. Scale bar=40μm. NS，no significance.


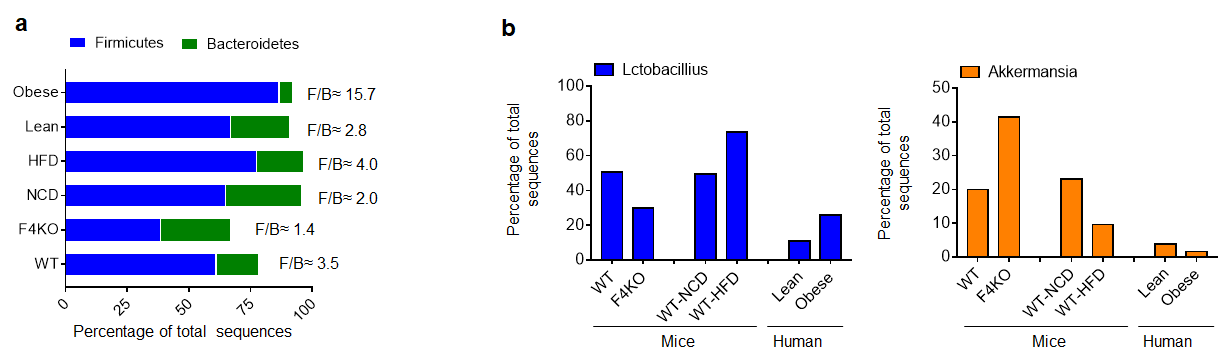


**Figure S7. The proportions in Phyla and Genus of gut microbiota.** (a)The proportions in Phyla of gut microbiota in FABP4^fl/fl^pvillin^CreT^ (F4KO) and FABP4^fl/fl^ (WT) mice, and mice fed on chow (WT-NCD) and high-fat diet (WT-HFD), or individuals with obesity (obese) and healthy individuals (lean). (b) The proportions in Genus and species of gut microbiota in FABP4^fl/fl^pvillin^CreT^ (F4KO) and FABP4^fl/fl^ (WT) mice, and mice fed on chow (WT-NCD) and high-fat diet (WT-HFD), or individuals with obesity (obese) and healthy individuals (lean).


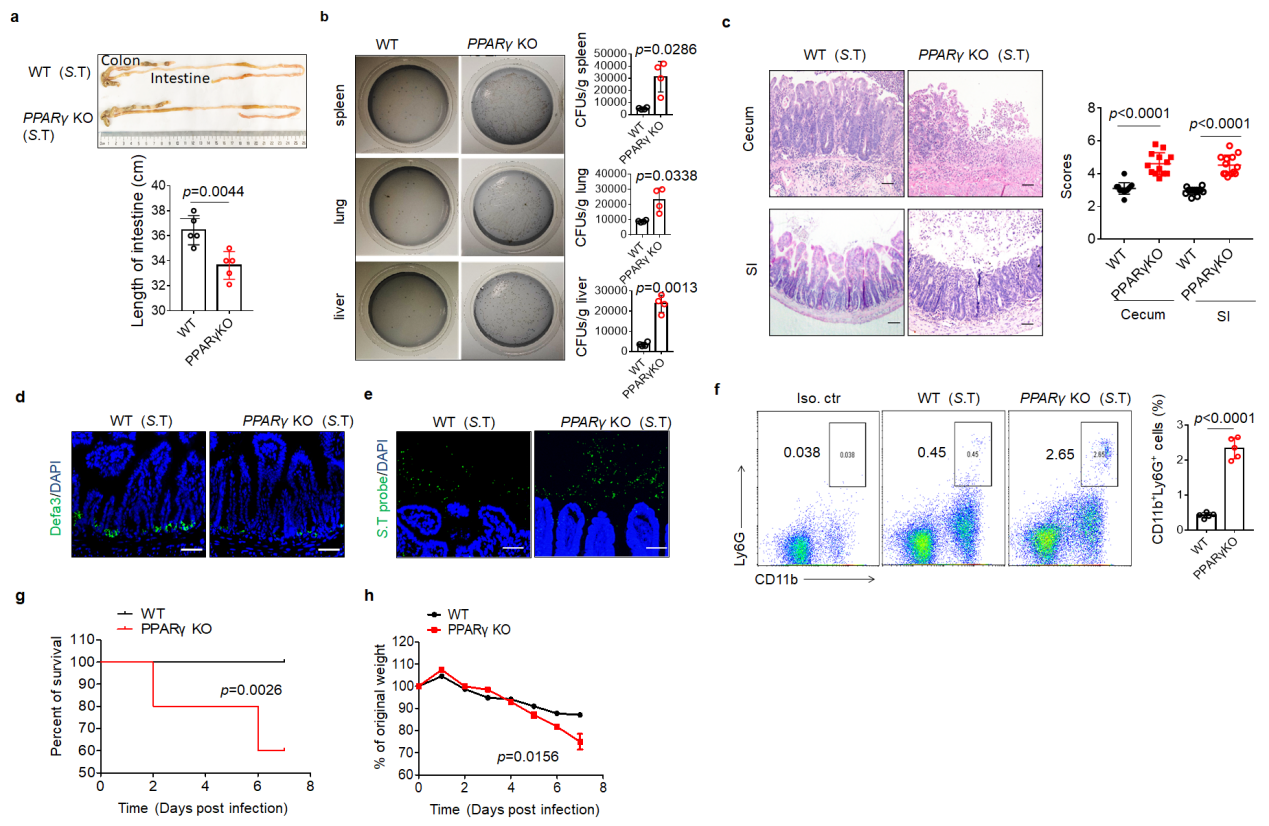


**Figure S8. *PPARγ* KO mice are more sensitive to *S.* T infection.** (a) Length of the intestines and colons of *PPARγ* KO and control WT mice after exposure to *S.*T (5×10^7^CFUs/mouse).(b) Representative bacteria plates images in lung, spleen and liver of *PPARγ* KO and control WT mice after exposure to *S.*T (5×10^7^CFUs/mouse). (c) H&E staining of cecum and small intestinal (SI) tissues of *PPARγ* KO and control WT mice after exposure to *S.*T (5×10^7^CFUs/mouse). Scale bars = 40 µm. (d) Immunostaining of Defa3 (green) in small intestinal tissues of *PPARγ* KO and control wild-type (WT*)* mice after exposure to *S.*T infection (5×10^7^CFUs/mouse). Scale bars=40 um. (e) Hybridization of *S.*T probes (green) in small intestinal tissues of *PPARγ* KO and control wild-type (WT*)* mice after exposing to *S.*T infection (5×10^7^CFUs/mouse). Scale bars=40 um. (f) Flow cytometry of CD11b^+^Ly6G^+^ cells in spleen tissues of *PPARγ* KO and WT mice after exposure to *S.*T (5×10^7^CFUs/mouse). (g) Survival rate of *PPARγ* KO and WT mice with chronic *S.*T infection (2×10^2^ CFUs/mouse). (h) Body weights of *PPARγ* KO and WT mice with chronic *S.*T infection (2×10^2^ CFUs/mouse). Student’s t test, mean ± SD in a, b and f; The Mann–Whitney U test in c. Analysis of variance test in h; Wilcoxon’s test in g; RE relative expression.


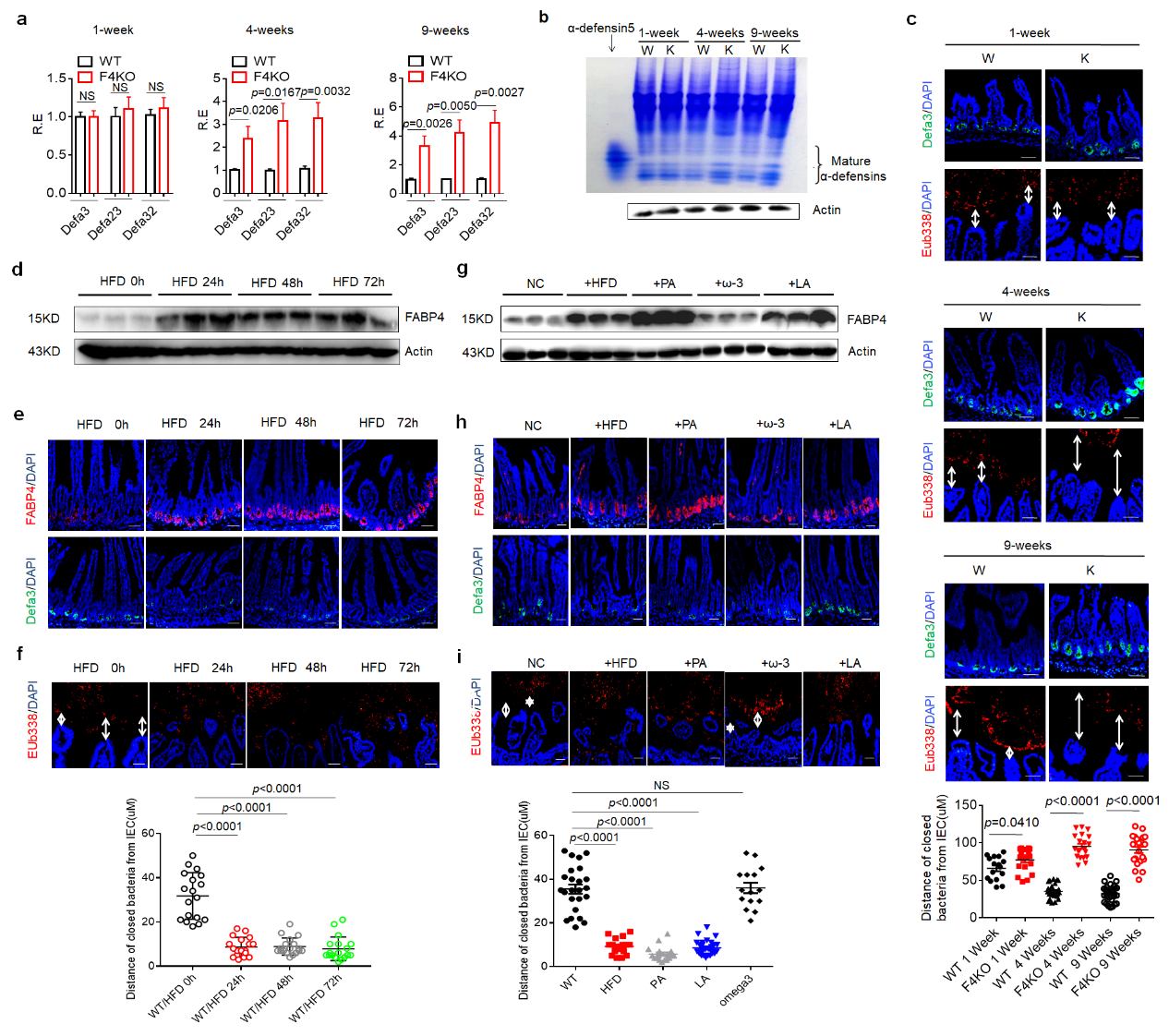


**Figure S9. HFD reduces expression of defesins in gut epithelial cells.** (a) QRT-PCR of Defa32/23/3 in the small intestinal tissues of 1 week, 4 weeks and 9 weeks old FABP4^fl/fl^pvillin^CreT^(F4KO) and FABP4^fl/fl^ (WT) mice. n=6. (b) AU-PAGE gel analysis of mature a-defensins in the small intestinal tissues of 1 week, 4 weeks and 9 weeks old FABP4^fl/fl^pvillin^CreT^(F4KO) and FABP4^fl/fl^ (WT) mice. Synthetic mature a-defensin 5, a positive control. Actin, a loading control. Pooled samples, n=5. (c) Imunostaining of Defa3 (upper) and hybridization of fluorescence Eub338 probe (lower) in the small intestinal tissues of 1 week, 4 weeks and 9 weeks old FABP4^fl/fl^pvillin^CreT^(F4KO) and FABP4^fl/fl^ (WT) mice. Quantification of the distance of closed bacteria from IEC (lower). Scale bars=40 um. (d) Immunobloting of FABP4 in small intestinal epithelial cells of FABP4^fl/fl^ mice fed HFD for 0 hr, 24 hrs, 48 hrs, and 72 hrs. Actin, a loading control. n=3. (e) Immunostaining of FABP4 (red) and Defa3 (green) in the small intestinal tissues of mice fed HFD at indicated time. Scale bar=40 um. (f) Hybridization of fluorescence Eub338 probe in small intestinal tissues and quantification of the distance of closed bacteria from IEC after exposure to HFD at indicated time. Scale bar=40 um. (g) Immunoblotting of FABP4 in small intestinal epithelial cells of FABP4^fl/fl^ mice after exposure to HFD, palmitic acid (PA), eicosapentaenoic acid (ω-3), linoleic acid (LA) for 2 days. Actin, a loading control. n=3. (h) Immunostaining of FABP4 (red) and Defa3 (green) in the ileums of FABP4^fl/fl^ mice after exposure to HFD, PA, ω-3, LA for 2 days. Scale bar=40 um

1. Hybridization of fluorescence Eub338 probe in small intestinal tissues of mice, and quantification of distance of closed bacteria from IEC after exposure to HFD, PA, ω-3, LA for 2 days. Scale bars=40 um. DAPI, nuclei. Student’s t test in a, mean ± SD; The Mann–Whitney U test in c, f and i.


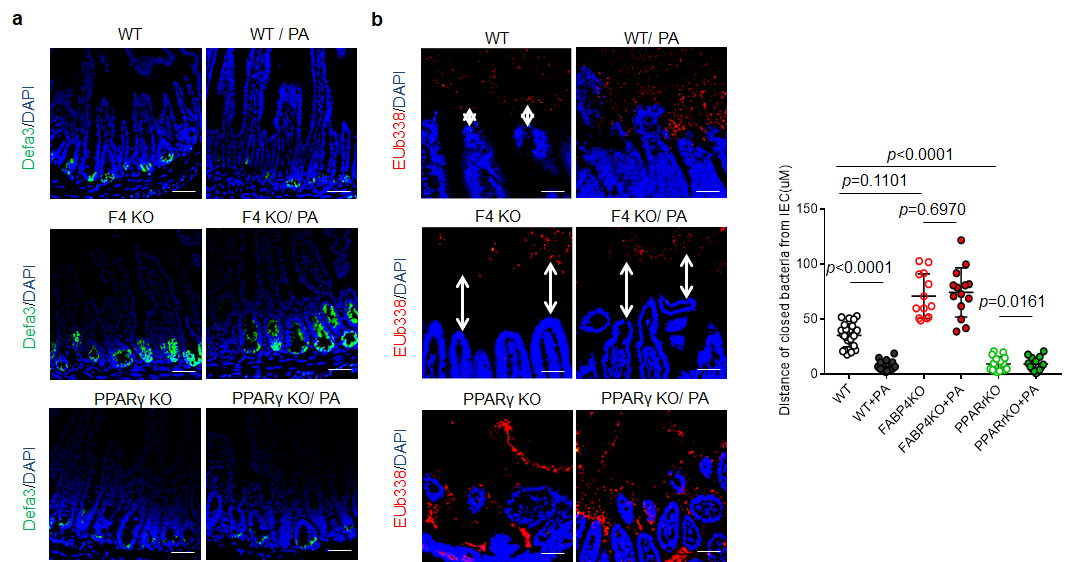


**Figure S10. HFD does not affect expression of defensins in FABP4^fl/fl^pvillin^CreT^ mice.** (a) Immunostaining of Defa3 in FABP4^fl/fl^(WT)，FABP4^fl/fl^pvillin^CreT^ (F4KO), and *PPARγ* KO mice small intestinal tissues of mice with or without PA for 2 days. Scale bar=40 um. (b) Hybridization of fluorescence EUb338 probe in the small intestinal tissues of mice with or without PA for 2 days. Scale bar=40 um. Scale bars=40 um; DAPI, nuclei; The Mann–Whitney U test in **c**. mean ± SD.


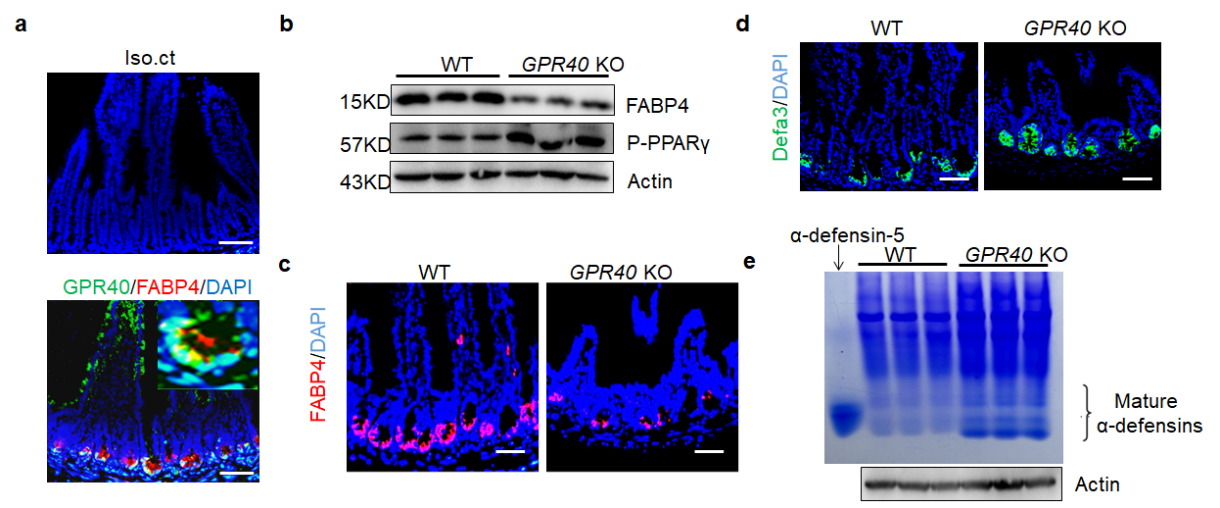


**Figure S11. *GRP40* deficiency reduces the expression of FABP4.** (a) Immunostaining of GRP40 (green) and FABP4 (red) in the epithelial cells of small intestines. Iso. ct, isotype control IgG. (b) Immunoblotting of FABP4 and P-PPARγ in small intestinal epithelial cells of *GRP40* KO and control WT mice. n=3. (c) Immunostaining of FABP4 (red) in small intestinal tissues of *GRP40* KO and control WT mice. (d) Immunostaining of Defa3 (green) in small intestinal tissues of *GRP40* KO and control WT mice. (e) AU-PAGE gel analyses of mature α-defensins in small intestinal tissues of *GRP40* KO and control WT mice. n=3. Actin, a loading control; DAPI, nuclei; Scar bar=40 um in a, c and d.


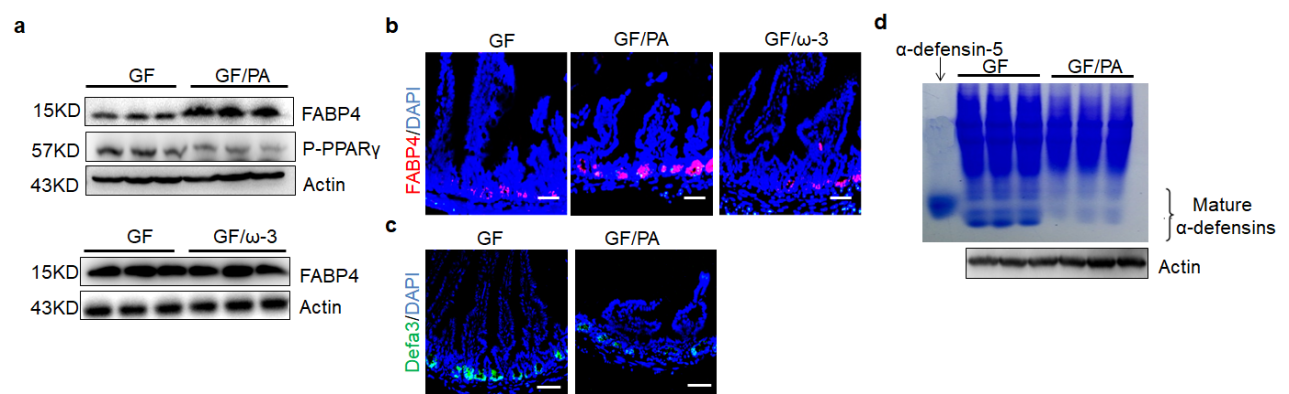


**Figure S12. HFD reduces the expression of defensins in germ-free mice.**

(a) Immunoblotting of FABP4 and P-PPARγ in small intestinal tissues of germ-free (GF) mice with (GF/PA or GF/ω-3) or without (GF) PA or ω-3. n=3. (b) Immunostaining of FABP4 (red) in small intestinal tissues of GF mice with (GF/PA or GF/ω-3) or without (GF) PA or ω-3 for 2days. (c) Immunostaining of Defa3 (green) in small intestinal tissues of GF mice with (GF/PA) or without (GF) PA. (d) AU-PAGE gel analyses of mature a-defensins in small intestinal tissues of GF mice with (GF/PA) or without (GF) PA. n=3. Actin, a loading control; DAPI, nuclei staining; Scar bar=40 um in b and c.


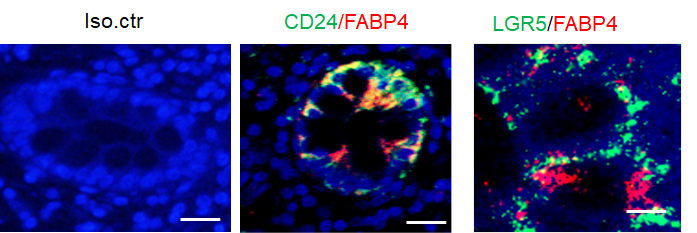


**Figure S13. Expression of FABP4 in human colonic Paneth-like cells.**

Immunostaining of CD24/FABP4 and LGR5/FABP4 in human colonic tissues. Scale bars= 5μm. Iso. ctr, isotypic control.

**Table S1. Reagents and oligoes used in this study.**

| **REAGENT or RESOURCE** | **SOURCE** | **IDENTIFIER** |
| --- | --- | --- |
| **Antibodies for Western blot** | | |
| β-Actin Antibody | Santa Cruz | Cat:sc-47778 RRID: AB_626632 |
| Anti-FABP4 Antibody | Abcam | Cat:ab92501 RRID: AB_10562486 |
| Anti-PPARγ Antibody | Abcam | Cat:ab209350 RRID: AB_2890099 |
| Anti-p-PPARγ Antibody | Thermo Fisher Scientific | Cat:PA5-104884 RRID: AB_2816357 |
| Anti-Ub Antibody | Abcam | Cat:ab134953 RRID: AB_2801561 |
| Anti-K48-Ub Antibody | Abcam | Cat:ab140601 RRID: AB_2783797 |
| Anti- K63-Ub Antibody | Abcam | Cat:ab179434 RRID: AB_2895239 |
| Goat anti-mouse IgG (H+L) -HRP antibody | ABclonal | Cat:AS003 RRID: AB_2769851 |
| Goat anti-rabbit IgG (H+L) -HRP antibody | ABclonal | Cat:AS014 RRID: AB_2769854 |
| Goat anti-mouse IgG (L) -HRP antibody | ABclonal | Cat:AS062 RRID: AB_2864056 |
| mouse anti-rabbit IgG (L) -HRP antibody | ABclonal | Cat:AS061 RRID: AB_2864055 |
| **Antibodies for flow cytometry analysis** | | |
| FITC anti-mouse F4/80 Antibody | Biolegend | Cat:123108 RRID: AB_893502 |
| PE anti-mouse CD206 Antibody | Biolegend | Cat: 141706 RRID: AB_10895754 |
| FITC anti-mouse CD11b Antibody | Thermo Fisher Scientific | Cat: 11-0112-82 RRID: AB_464935 |
| PE anti-mouse Ly6G Antibody | BD Bioscience | Cat:551461 RRID: AB_394208 |
| APC anti-mouse CD11c Antibody | Biolegend | Cat: 117310 RRID: AB_ 313779 |
| FITC-Lysozyme Antibody | Novus | Cat: NBP2-61118 |
| **Antibodies for Immunostaining** |  |  |
| Anti-Human/Mouse FABP4 Antibody | Abcam | Cat:ab92501 RRID: AB_10562486 |
| Anti-Human/Mouse p-PPARγ Antibody | Bioss | Cat:bs-4888R |
| Anti-Human/Mouse Lysozyme Antibody | Abcam | Cat:ab108508 RRID: AB_10861277 |
| Anti-Human/Mouse LGR5 Antibody | Thermo Fisher Scientific | Cat:MA5-25644 RRID: AB_2723318 |
| Anti-Mouse DEFA3 Antibody | Thermo Fisher Scientific | Cat:PA5-103122 RRID: AB_2852492 |
| Anti-Mouse Ki67 Antibody | Abcam | Cat:ab15580 RRID: AB_443209 |
| Anti-Human CD24 Antibody | Thermo Fisher Scientific | Cat:MA5-11828 RRID: AB_10983158 |
| Anti-Human DEFA5 Antibody | Thermo Fisher Scientific | Cat:MA1-46026 RRID: AB_2091702 |
| FITC-Goat Anti-Rat IgG (H+L) Antibody | Proteintech | Cat:SA00003-11 RRID: AB_2890989 |
| TRITC-Goat Anti-Rat IgG (H+L) Antibody | Proteintech | Cat:SA00007-7 RRID: AB_2890953 |
| Alexa Fluor 488 Goat Anti-Mouse IgG(H+L) Antibody | Proteintech | Cat:SA00003-1 RRID: AB_2890896 |
| Alexa Fluor 594 Goat Anti-Mouse IgG(H+L) Antibody | Proteintech | Cat:SA00013-3 RRID: AB_2797133 |
| Alexa Fluor 488 Goat Anti-Rabbit IgG(H+L) Antibody | Proteintech | Cat:SA00003-2 RRID: AB_2890897 |
| TRITC- Goat Anti-Rabbit IgG(H+L) Antibody | Proteintech | Cat:SA00007-2 RRID: AB_2889939 |
| TRITC-Rabbit Anti-Goat IgG (H+L) Antibody | Proteintech | Cat:SA00007-4 RRID: AB_2890952 |
| FITC-Rabbit Anti-Goat IgG (H+L) Antibody | Bioss | Cat:bs-0294R RRID: AB_10893518 |
| **Fluorescence probe sequences** | | |
| Eub338 | Cy3-GCT GCC TCC CGT AGG AGT | |
| *Salmonella* Typhimurium | FAM-CTCTTTCGTCTGGCATTATCGATCAGTACCA | |
| **Oligonucleotides for qRT-PCR** | | |
| Murine GAPDH FW | BGI | 5’-TCAACGGCACAGTCAAGG-3’ |
| Murine GAPDH Rev | BGI | 5’-TACTCAGCACCGGCCTCA-3’ |
| Murine TNFa FW | BGI | 5’-CCAGACCCTCACACTCAGATCA-3’ |
| Murine TNFa Rev | BGI | 5’-GTAGACAAGGTACAACCCATCG-3’ |
| Murine IL-6 FW | BGI | 5’-ACAACCACGGCCTTCCCTACTT-3’ |
| Murine IL-6 Rev | BGI | 5’-TTTCTCATTTCCACGATTTCCC-3’ |
| Murine IL-18 FW | BGI | 5’-ATGGCTGCCATGTCAGAAG-3’ |
| Murine IL-18 Rev | BGI | 5’-TAACTTTGATGTAAGTTAGTGAGAG-3’ |
| Murine IL-1β FW | BGI | 5’-TTGACGGACCCCAAAAGATG-3’ |
| Murine IL-1β Rev | BGI | 5’-AGAAGGTGCTCATGTCCTCA-3’ |
| Murine GM-csf FW | BGI | 5’-GGGGGCAGTATGTCTGGTAG-3’ |
| Murine GM-csf Rev | BGI | 5’-GCTCCAGGGACTTAAGCAGG-3’ |
| Murine DEFA3 FW | BGI | 5’-ACACTAGTCCTCCTCTCTGCC-3’ |
| Murine DEFA3 Rev | BGI | 5’-AGAGCCTTCTGGGTCTCCAA-3’ |
| Murine DEFA23 FW | BGI | 5’-TCCTCTCTGCCCTCATCCTG-3’ |
| Murine DEFA23 Rev | BGI | 5’-CACAGCCTGGTCCTCTTTCC-3’ |
| Murine DEFA32 FW | BGI | 5’-CGACTTTTGTTCTGCTGCCG-3’ |
| Murine DEFA32 Rev | BGI | 5’-AGCCTCAGAGCTGATGGTTG-3’ |
| Murine MMP-7 FW | BGI | 5’-GCTCACCCTGTTCTGCTTTGT-3’ |
| Murine MMP-7 Rev | BGI | 5’-ATTCTGAGCCTGTTCCCACTG-3’ |
| Murine DEFβ1 FW | BGI | 5’-TCACATCCTCTCTGCACTCTG-3’ |
| Murine DEFβ1 Rev | BGI | 5’-TCCAAGACTTGTGAGAATGCCA-3’ |
| Murine DEFβ2 FW | BGI | 5’-TGAGTGCCCTTTCTACCAGC-3’ |
| Murine DEFβ2 Rev | BGI | 5’-TGTGGCAGTGGTCAAGTTCT-3’ |
| Murine DEFβ3 FW | BGI | 5’-TCTGTTTGCATTTCTCCTGGTG-3’ |
| Murine DEFβ3 Rev | BGI | 5’-TCTGACGAGTGTTGCCAATG-3’ |
| Murine Reg3α FW | BGI | 5’-GCTCCCACTGCTATGCCTTA-3’ |
| Murine Reg3α Rev | BGI | 5’-AAAGGAAGCCTCACCTCCAC-3’ |
| Murine Reg3γ FW | BGI | 5’-TTCCTGTCCTCCATGATCAAA -3’ |
| Murine Reg3γ Rev | BGI | 5’-CATCCACCTCTGTTGGGTTC-3’ |
| Murine FABP4 FW | BGI | 5’-GGATTTGGTCACCATCCGGT-3’ |
| Murine FABP4 Rev | BGI | 5’-TTCCATCCCACTTCTGCACC-3’ |
| Murine LGR-5 FW | BGI | 5’-CTGCCAAATCGTTGGCTTCC-3’ |
| Murine LGR-5 Rev | BGI | 5’-GCAAGGGGATTGTGGCAATG-3’ |
| Murine Lysozyme FW | BGI | 5’-ATGGAATGGATGGCTACCGT-3’ |
| Murine Lysozyme Rev | BGI | 5’-GGGGTTTTGCCATCATTACACC-3’ |
| Murine Saa1 FW | BGI | 5’-ATGTGGCGAGCCTACACTG-3’ |
| Murine Saa1 Rev | BGI | 5’-TGGTCAGCAATGGTGTCCTC-3’ |
| Murine Pla2g2a FW | BGI | 5’-CAGACCGGTGCTGTGTTACT-3’ |
| Murine Pla2g2a Rev | BGI | 5’-GTTCCGGGCGAAACATTCAG-3’ |
| Human FABP4 FW | BGI | 5’-CCTTAGATGGGGGTGTCCTG-3’ |
| Human FABP4 Rev | BGI | 5’-GCCTTTCATGACGCATTCCA-3’ |
| Human DEFA5 FW | BGI | 5’-GGCTACAACCCAGAAGCAGT-3’ |
| Human DEFA5 Rev | BGI | 5’-CGGCCACTGATTTCACACAC-3’ |
| Human GAPDH FW | BGI | 5’-GTCAAGGCTGAGAACGGGAA-3’’ |
| Human GAPDH Rev | BGI | 5’-AAATGAGCCCCAGCCTTCTC-3’ |
| **Oligonucleotides for CHIP-PCR** | | |
| Murine DEFA32-FW | BGI | 5’-GACCAAAGATGTAAGAGACC-3’ |
| Murine DEFA32-RW | BGI | 5’-TTAAAGTGTAACATGGGAAG-3’ |
| **siRNAs and FABP4 plasmid** | | |
| Human PPARγsiRNA | Santa Cruz | Cat:sc-29455 |
| Human FABP4 siRNA | Santa Cruz | Cat:sc-43592 |
| Human FABP4 plasmid | HANBIO | Cat:HH20210723GX-SI01 |
| **Bacteria strain** |  |  |
| *Salmonella* Typhimurium | ATCC14028 | |
| **Chemicals** | | |
| Dextran sulfate sodium salt (DSS) | MP Biomedicals | Cat: 160110 |
| High-fat diet | Research Diets | Cat: D12492 |
| Streptomycin | MCE | Cat: HY-B0472 |
| Palmitic Acid | MCE | Cat: HY-N0830 |
| Pioglitazone | MCE | Cat: HY-13956 |
| ω-3 | MCE | Cat: HY-B0660 |
| Linoleic Acid | MCE | Cat: HY-N0729 |
| MacConkey Agar | Oxoid | Cat: CM0007B |
| LB Broth | Sigma-Aldrich | Cat: L3522 |
| Vancomycine | Sigma-Aldrich | Cat: V2002 |
| Ampicillin | Sigma-Aldrich | Cat: A5354 |
| Neomycin sulfate | Sigma-Aldrich | Cat: N6386 |
| Metronidazole | Sigma-Aldrich | Cat: M1547 |
| Trizol | Life Technologies | Cat: 15596026 |
| FBS | Gibco | Cat:10099141 |
| DAPI | SouthemBiotech | Cat:0100-20 |
| Collagenase IV | Sigma-Aldrich | Cat: C5138 |
| Dnase I | Solarbio | Cat: D8071 |
| TrypLE^TM^ Express | Gibco | Cat: 12604013 |
| Dispase | Corning | Cat: 354235 |
| DMEM | Gibco | Cat:11965118 |
| HBSS | Gibco | Cat:14170161 |
| Pecoll | Solarbio | Cat: P8370 |
| PMA | Sigma-Aldrich | Cat: 79346 |
| Ionomycin | Sigma-Aldrich | Cat: 19657 |
| GolgiStop | BD Biosciences | Cat: 554724 |
| ProteinA/G Magnetic Beads | MCE | Cat:HY-K02 |
| EDTA | Sigma-Aldrich | Cat: 798681 |
| Gentle Cell Dissociation Reagent | STEMCELL | Cat: 07174 |
| Y-27632 | MCE | Cat: HY-10583 |
| IntestiCult OGM Mouse Kit | STEMCELL | Cat: 06005 |
| IntestiCult OGM Human Kit | STEMCELL | Cat: 06010 |
| Cell strainer | Biosharp | Cat: BS-70-XBS |
| Matrigel MatriX | Corning | Cat:356231 |
| DMEM/F12 | STEMCELL | Cat:36254 |
| **Critical Commercial Assays** | | |
| Human DEFA5 ELISA Kit | Cloud-clone | Cat:SEB912Hu |
| Mouse DEFA3 ELISA Kit | Cloud-clone | Cat:SEE135Mu |
| QIAquick PCR Purification Kit | Qiagen | Cat:28104 |
| QuantiTect SYBR Green PCR Master Mix | Qiagen | Cat:208052 |
| Cell stimulation cocktail | ebioscience | Cat: 00-4975-03 |
| Permeabilization Buffer | Thermo Fisher | Cat: 00-8333-56 |
| ECL chemiluminescence | Absin | Cat: abs920 |
| Protease Inhibitor Cocktail | Sigma-Aldrich | Cat: P8340 |
